# Role of Resveratrol in Transmitochondrial AMD RPE Cells

**DOI:** 10.3390/nu12010159

**Published:** 2020-01-06

**Authors:** Sonali Nashine, Anthony B. Nesburn, Baruch D. Kuppermann, Maria Cristina Kenney

**Affiliations:** 1 Department of Ophthalmology, Gavin Herbert Eye Institute, University of California Irvine, Irvine, CA 92697, USA; snashine@uci.edu (S.N.); anesburn@uci.edu (A.B.N.); bdkupper@uci.edu (B.D.K.); 2 Cedars-Sinai Medical Center, Los Angeles, CA 92697, USA; 3 Department of Pathology and Laboratory Medicine, University of California Irvine, Irvine, CA 92697, USA

**Keywords:** resveratrol, nutraceutical, stilbenoid, phytoalexin, age-related macular degeneration, AMD, macular degeneration, retina, retinal pigment epithelial cells, RPE, cybrids, over-the-counter nutritional supplements

## Abstract

Resveratrol is a phytoalexin, stilbenoid compound with antioxidant properties attributable to its bioactive *trans*-resveratrol content. This study characterized the effects of over-the-counter (OTC) resveratrol nutritional supplements and a HPLC-purified resveratrol formulation, in human transmitochondrial age-related macular degeneration (AMD) retinal pigment epithelial (RPE) patient cell lines. These cell lines, which were created by fusing blood platelets obtained from dry and wet AMD patients with mitochondria-deficient (Rho0) ARPE-19 cells, had identical nuclei (derived from ARPE-19 cells) but different mitochondria that were derived from AMD patients. After resveratrol treatment, the levels of cell viability and reactive oxygen species production were measured. Results demonstrated that treatment with different resveratrol formulations improved cell viability and decreased reactive oxygen species generation in each AMD patient cell line. Although further studies are required to establish the cytoprotective potential of resveratrol under different physiological conditions, this novel study established the positive effects of OTC resveratrol supplements in macular degeneration patient cybrid cell lines in vitro.

## 1. Introduction

Resveratrol is a biologically active stilbenoid i.e., a plant polyphenolic compound, and is commonly found in grapes’ (*Vitis vinifera*) skin and seeds, red and white wine, Japanese knotweed (*Polygonum cuspidatum*), blueberries, cranberries, bilberries, cocoa, pistachios, and peanuts. Compared to the other varieties of wines, red wine has the highest resveratrol content because it is produced by crushing the grapes and leaving the skins in contact with the juice throughout the fermentation process. The Malbec grapes are thicker skinned and therefore have higher concentration of resveratrol compared to the other varieties of red wine grapes [1].

Resveratrol is a phytoalexin, which is synthesized de novo by numerous plants, including vines, in response to pathogen infection. Chemically, resveratrol occurs in two isomeric forms namely *trans*-resveratrol and cis-resveratrol. Dihydro-resveratrol is a metabolite of both cis- and trans-resveratrol having a different molecular weight. Trans-resveratrol i.e., 3,5,41-trihydroxy-*trans-*stilbene, is the predominant bioactive form, which exhibits a broad spectrum of pharmacological properties. The anti‑angiogenic activity of resveratrol has been widely studied and the role of resveratrol in attenuation of VEGF-mediated angiogenesis has been well‑established in recent studies [2,3].

Resveratrol extracted from grapes, red wine, and *Polygonum cuspidatum* are available as over‑the-counter (OTC) dietary supplements in pharmacies all over the US. These supplements contain varying concentrations of *trans*-resveratrol, anywhere from 1 mg to 1000 mg, and therefore might differ in their efficacies in patients. Orally administered resveratrol is absorbed in the gut, undergoes bioconversion by gut microbiota, and is excreted in urine. Resveratrol’s major metabolic reactions involve formation of conjugates with glucuronic acid and sulfate. Since it is rapidly metabolized, resveratrol concentration is relatively low in plasma and is barely detectable in the blood. This paradox i.e., high bioactivity and low bioavailability of resveratrol, is still a matter of debate because it is speculated that resveratrol cannot exert a multitude of effects unless it has high systemic availability [4,5]. The resveratrol formulations tested in this study had varying concentrations of the active *trans*-resveratrol.

Despite being the primary cause of vision loss in the US, age‑related macular degeneration (AMD) has limited treatment options available, which include AREDS formulations and frequent intravitreal injections of anti-VEGF drugs [6]. Since resveratrol has been previously shown to reduce oxidative stress in RPE cells and is known to exert anti-angiogenic effects [7], screening new OTC resveratrol formulations in AMD RPE cell lines was relevant. The goal of the current study was to investigate and compare the effects of various OTC resveratrol formulations in AMD RPE transmitochondrial cell lines in order to identify the most effective OTC resveratrol formulations. These AMD RPE cybrid cell lines, which were created by fusing mitochondria-rich blood platelets from AMD patients with mitochondria-deficient Rho0 ARPE-19 cells, had the same nuclear content but mitochondria derived from different AMD patients.

We found that the six different formulations of resveratrol tested in this study produced higher levels of cellular viability and decreased free radical production in each AMD patient cell line, although to varying degrees. The variability in effects between the formulations could be attributed to either: (a) The ingredients and *trans*-resveratrol content of each of the formulations and/or (b) to the patient-specific mitochondrial differences. This study provides in vitro evidence suggesting potential beneficial effects of using over-the-counter dietary resveratrol supplements as a nutraceutical for both dry AMD and wet AMD patients. However, further validation studies are required to understand the mechanisms of action for the tested resveratrol formulations.

## 2. Methods

### 2.1. Human Subjects

The Institutional review board of the University of California Irvine approved research with human subjects (Approval UCI IRB#2003–3131). All AMD patients recruited in this study (Table 1) provided informed consent and clinical investigations were performed according to the tenets of the Declaration of Helsinki.

### 2.2. Cell Culture

ARPE-19 cells, transmitochondrial normal RPE cybrid, and transmitochondrial AMD RPE cybrid cells were used in this study. Transmitochondrial cybrids were created by fusing mitochondrial DNA-deficient APRE-19 (Rho0) cell line with platelets isolated from either wet or dry AMD patients. Peripheral blood (10 mL) was collected via venipuncture in tubes containing 10 mM EDTA. Blood platelets were isolated by a series of centrifugation steps, in tubes containing 3.2% sodium citrate, and final pellets were suspended in Tris buffered saline. Cybrids were created by polyethylene glycol fusion of platelets with Rho0 ARPE-19 cells in medium containing uridine. After growing the cybrid cells in uridine-containing medium for 2 weeks, the medium was replaced with regular culture medium. Mitochondrial DNA genetic profiles of each cybrid cell line were confirmed using PCR, restriction enzyme digestion, and sequencing. All transmitochondrial cybrids were grown in DMEM/Ham’s F12 1:1 cell culture medium containing 24 mM sodium bicarbonate, 10% dialyzed fetal bovine serum, and 1.0 mM sodium pyruvate. [8]. Passage 5 cells were used for all experiments (*n* = 13). The cells were seeded at a density of 5000–10,000 cells per well and they reached confluence at the end of the experiment. Allelic discrimination and Sanger Sequencing were used to verify that the mtDNA within the newly created cybrids and the original AMD subject were the same and thereby confirm the status of the cybrid.

### 2.3. Resveratrol Treatment

Resveratrol formulations were used at a concentration of 1000 μM for all experiments. Dimethyl sulfoxide (DMSO) was used as an initial solvent. Resveratrol was subsequently dissolved in culture media for cell treatment. Given below are the names and ingredients of the resveratrol brands tested in the current study:

Resveratrol Brand 1 (B1): Purified resveratrol (Sigma-Aldrich, St. Louis, MO, USA): ≥99% HPLC-purified *trans*-resveratrol powder.

Resveratrol Brand 2 (B2): Capsules; resveratrol Polyphenol Complex (600 mg) containing 250 mg of *trans*-resveratrol, organic French whole red wine grape (*Vitis vinifera*) (skin, seeds, fruit, stem, vine), certified organic muscadine whole red grape (*Vitis rotundifolia*) (skin and seed); Quercetin (as quercetin dihydrate)—100 mg. Other ingredients: Vegetarian capsule (vegetable cellulose), rice bran, silica.

Resveratrol Brand 3 (B3): Capsules; resveratrol Complex containing 100 mg *trans*‑resveratrol. Proprietary resveratrol Complex (300 mg)—*Polygonum cuspidatum* root extract, grape seed (*Vitis vinifera*) extract, grape skin (*Vitis vinifera*) extract, provides 100 mg *trans*-resveratrol. Other ingredients: Maltodextrin, Gelatin, Dicalcium Phosphate, Stearic acid, Microcrystalline cellulose, Vegetable Magnesium Stearate, Croscarmellose Sodium, and Silica.

Resveratrol Brand 4 (B4): Capsules; red wine complex (500 mg)—grape seed extract, *trans*‑resveratrol (from *Polygonum cuspidatum* root extract), red wine extract. Other Ingredients: Dicalcium phosphate, gelatin, magnesium stearate, silicon dioxide.

Resveratrol Brand 5 (B5): Capsules; resveratrol (*Polygonum cuspidatum* root)—1000 mg; other ingredients: Gelatin and Rice Powder.

Resveratrol Brand 6 (B6): Capsules; resveratrol (*Polygonum cuspidatum* root)—1000 mg; other ingredients: Vegetable Cellulose (capsule), Rice flour.

### 2.4. Cell Viability Assay

Cell viability was measured using MTT (3-(4,5-dimethylthiazol-2-yl)-2, 5-diphenyltetrazolium bromide) assay (Cat. # 30006, Biotium, Fremont, CA, USA). Cells were plated in 96-well tissue culture plates and treated with resveratrol for 48 h. Cells were incubated with MTT reagent at 37 °C for 1 h, followed by addition of DMSO. Signal absorbance was measured at 570 nm and background absorbance was measured at 630 nm. Normalized absorbance values were obtained by subtracting background absorbance from signal absorbance. The colorimetric signal obtained was proportional to the cell number.

### 2.5. Reactive Oxygen Species (ROS) Assay

To quantitate ROS levels, the cell-permeant H2DCFDA (2′,7′-dichlorodihydrofluorescein diacetate) was used as an indicator for ROS in cells. Stock solution of 5mM H2DCFDA was prepared in DMSO. Stock solution was then diluted in Dulbecco’s phosphate buffered saline (DPBS) to obtain a working concentration of 10 μM. Cells were plated in 96-well tissue culture plates followed by treatment with resveratrol. Then, 10 μM H2DCFDA solution was added to cells and incubated for 30 min at 37 °C. H2DCFDA was then replaced with DPBS. Fluorescence, which was measured at excitation 492 nm and emission 520 nm, was proportional to ROS levels in cells.

### 2.6. Statistical Analysis

Non-parametric Mann–Whitney tests (GraphPad Prism 5.0; GraphPad Software, San Diego, CA, USA) were performed to analyze data between groups. *p* values ≤ 0.05 were considered statistically significant.

## 3. Results

### 3.1. Effects of Resveratrol Brand 1 (B1)

#### 3.1.1. Cell Viability

**Figure 1:**
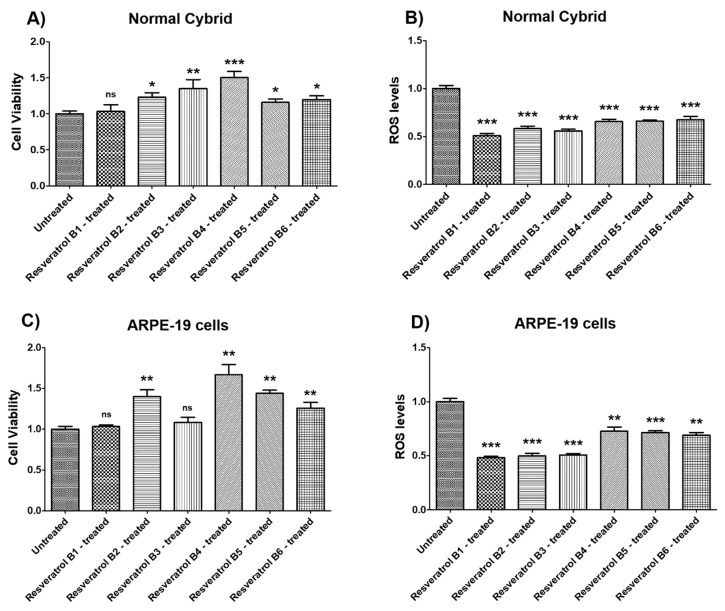
Effects of resveratrol formulations on cell viability and reactive oxygen species (ROS) levels in normal cybrids (**A**,**B**) and in ARPE-19 cell lines (**C**,**D**). Bar 1—untreated cells; Bar 2—resveratrol B1-treated cells; Bar 3—resveratrol B2-treated cells; Bar 4—resveratrol B3-treated cells; Bar 5—resveratrol B4-treated cells; Bar 6—resveratrol B5-treated AMD cells; Bar 7—resveratrol B6-treated cells. Data are presented as mean ± SEM; * *p* < 0.05; ** *p* < 0.01; *** *p* < 0.001; ns = non‑significant.

Resveratrol B1 i.e., the ≥99% HPLC-purified, *trans*‑resveratrol powder, caused no significant difference in cell viability in normal (NL) cybrid (*p* = 0.7748) (Figure 1A, Table 2a) or in wildtype ARPE‑19 cells (*p* = 0.5476) (Figure 1C, Table 2c).

**Figure 2:**
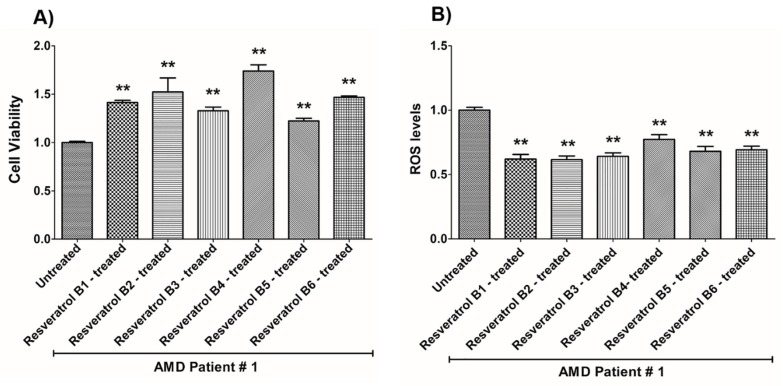
Effects of resveratrol formulations on cell viability (**A**) and ROS levels (**B**) in AMD patient #1. Bar 1—untreated AMD cells; Bar 2—resveratrol B1-treated AMD cells; Bar 3—resveratrol B2-treated AMD cells; Bar 4—resveratrol B3-treated AMD cells; Bar 5—resveratrol B4-treated AMD cells; Bar 6—resveratrol B5-treated AMD cells; Bar 7—resveratrol B6-treated AMD cells. Data are presented as mean ± SEM; ** *p* < 0.01.

**Figure 3:**
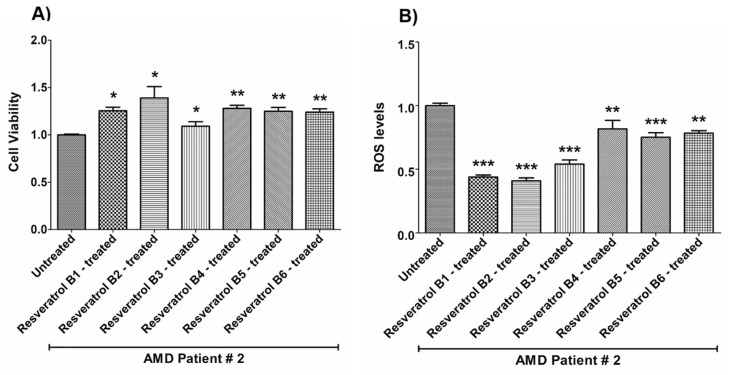
Effects of resveratrol formulations on cell viability (**A**) and ROS levels (**B**) in AMD patient #2. Bar 1—untreated AMD cells; Bar 2—resveratrol B1-treated AMD cells; Bar 3—resveratrol B2-treated AMD cells; Bar 4—resveratrol B3-treated AMD cells; Bar 5—resveratrol B4-treated AMD cells; Bar 6—resveratrol B5-treated AMD cells; Bar 7—resveratrol B6-treated AMD cells. Data are presented as mean ± SEM; * *p* < 0.05; ** *p* < 0.01; *** *p* < 0.001.

**Figure 4:**
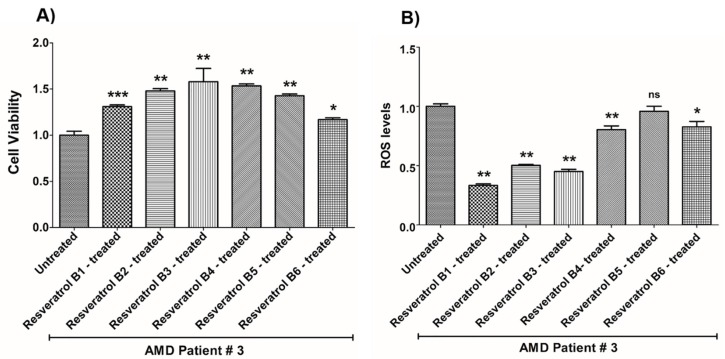
Effects of resveratrol formulations on cell viability (**A**) and ROS levels (**B**) in AMD patient #3. Bar 1—untreated AMD cells; Bar 2—resveratrol B1-treated AMD cells; Bar 3—resveratrol B2-treated AMD cells; Bar 4—resveratrol B3-treated AMD cells; Bar 5—resveratrol B4-treated AMD cells; Bar 6—resveratrol B5-treated AMD cells; Bar 7—resveratrol B6-treated AMD cells. Data are presented as mean ± SEM; * *p* < 0.05; ** *p* < 0.01; *** *p* < 0.001; ns = non-significant.

**Figure 5:**
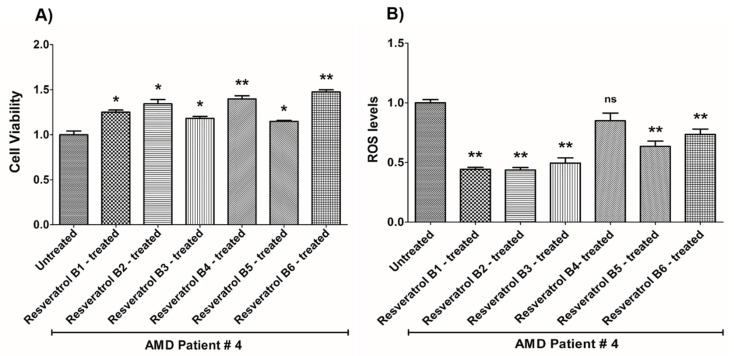
Effects of resveratrol formulations on cell viability (**A**) and ROS levels (**B**) in AMD patient #4. Bar 1—untreated AMD cells; Bar 2—resveratrol B1-treated AMD cells; Bar 3—resveratrol B2-treated AMD cells; Bar 4—resveratrol B3-treated AMD cells; Bar 5—resveratrol B4-treated AMD cells; Bar 6—resveratrol B5-treated AMD cells; Bar 7—resveratrol B6-treated AMD cells. Data are presented as mean ± SEM; * *p* < 0.05; ** *p* < 0.01; ns = non-significant.

**Figure 6:**
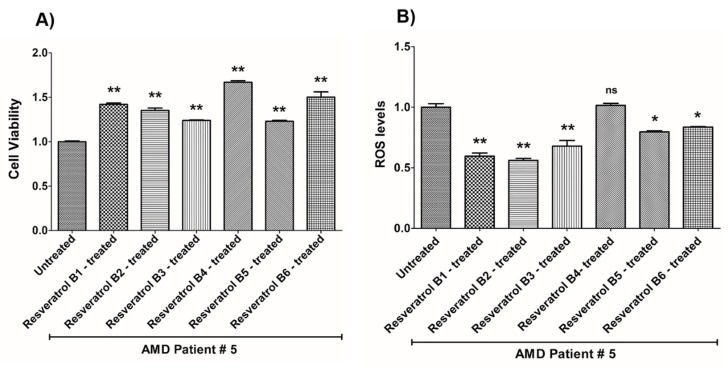
Effects of resveratrol formulations on cell viability (**A**) and ROS levels (**B**) in AMD patient #5. Bar 1—untreated AMD cells; Bar 2—resveratrol B1-treated AMD cells; Bar 3—resveratrol B2-treated AMD cells; Bar 4—resveratrol B3-treated AMD cells; Bar 5—resveratrol B4-treated AMD cells; Bar 6—resveratrol B5-treated AMD cells; Bar 7—resveratrol B6-treated AMD cells. Data are presented as mean ± SEM; * *p* < 0.05; ** *p* < 0.01; ns = non‑significant.

**Figure 7:**
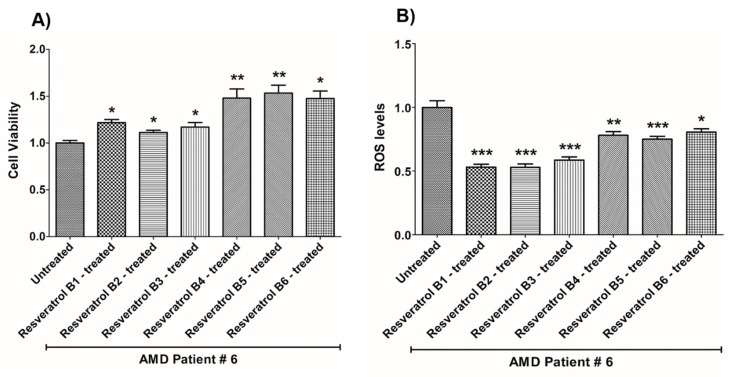
Effects of resveratrol formulations on cell viability (**A**) and ROS levels (**B**) in AMD patient #6. Bar 1—untreated AMD cells; Bar 2—resveratrol B1-treated AMD cells; Bar 3—resveratrol B2-treated AMD cells; Bar 4—resveratrol B3-treated AMD cells; Bar 5—resveratrol B4-treated AMD cells; Bar 6—resveratrol B5-treated AMD cells; Bar 7—resveratrol B6-treated AMD cells. Data are presented as mean ± SEM; * *p* < 0.05; ** *p* < 0.01; *** *p* < 0.001.

**Figure 8:**
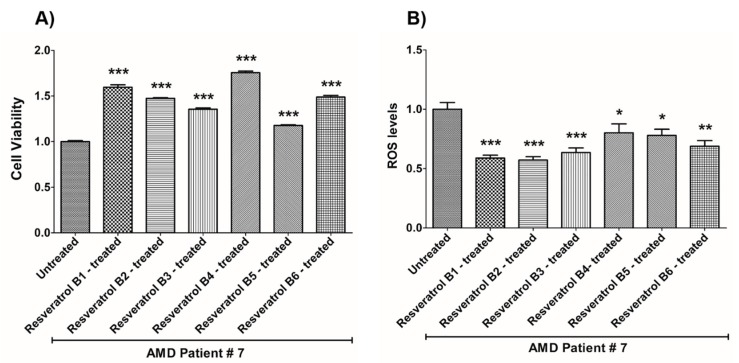
Effects of resveratrol formulations on cell viability (**A**) and ROS levels (**B**) in AMD patient #7. Bar 1—untreated AMD cells; Bar 2—resveratrol B1-treated AMD cells; Bar 3—resveratrol B2-treated AMD cells; Bar 4—resveratrol B3-treated AMD cells; Bar 5—resveratrol B4-treated AMD cells; Bar 6—resveratrol B5-treated AMD cells; Bar 7—resveratrol B6-treated AMD cells. Data are presented as mean ± SEM; * *p* < 0.05; ** *p* < 0.01; *** *p* < 0.001.

**Figure 9:**
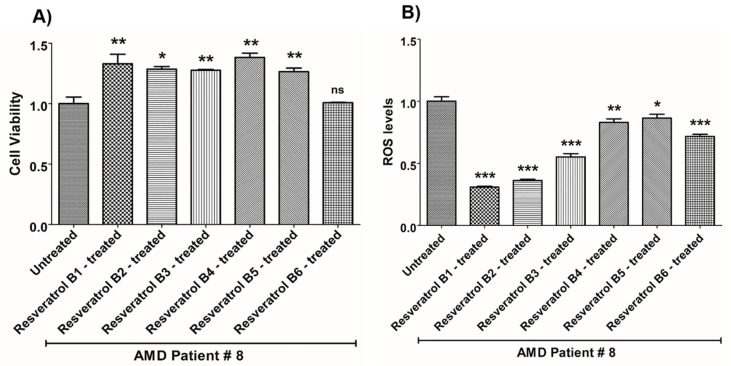
Effects of resveratrol formulations on cell viability (**A**) and ROS levels (**B**) in AMD patient #8. Bar 1—untreated AMD cells; Bar 2—resveratrol B1-treated AMD cells; Bar 3—resveratrol B2-treated AMD cells; Bar 4—resveratrol B3-treated AMD cells; Bar 5—resveratrol B4-treated AMD cells; Bar 6—resveratrol B5-treated AMD cells; Bar 7—resveratrol B6-treated AMD cells. Data are presented as mean ± SEM; * *p* < 0.05; ** *p* < 0.01; *** *p* < 0.001; ns = non-significant.

**Figure 10:**
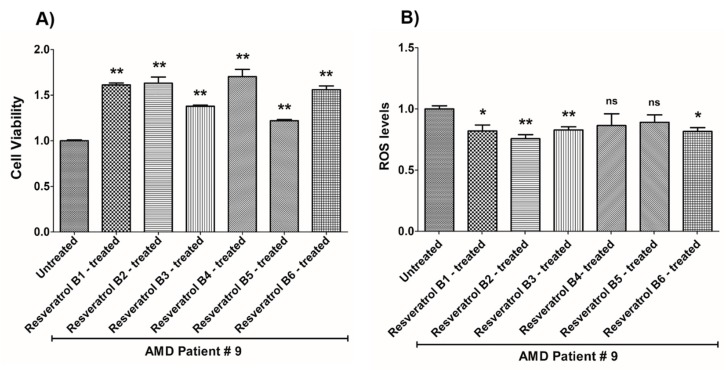
Effects of resveratrol formulations on cell viability (**A**) and ROS levels (**B**) in AMD patient #9. Bar 1—untreated AMD cells; Bar 2—resveratrol B1-treated AMD cells; Bar 3—resveratrol B2-treated AMD cells; Bar 4—resveratrol B3-treated AMD cells; Bar 5—resveratrol B4-treated AMD cells; Bar 6—resveratrol B5-treated AMD cells; Bar 7—resveratrol B6-treated AMD cells. Data are presented as mean ± SEM; * *p* < 0.05; ** *p* < 0.01; ns = non-significant.

**Figure 11:**
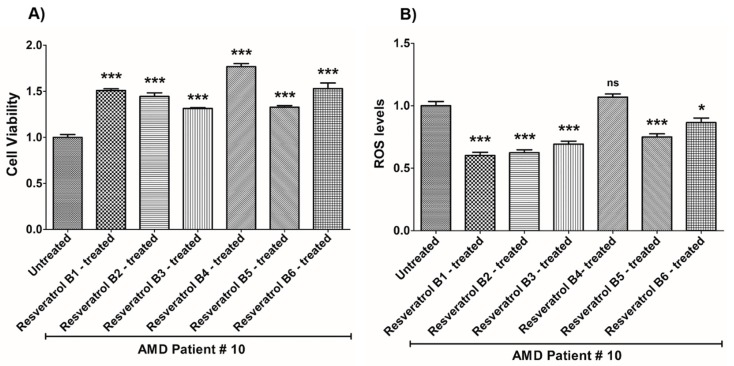
Effects of resveratrol formulations on cell viability (**A**) and ROS levels (**B**) in AMD patient #10. Bar 1—untreated AMD cells; Bar 2—resveratrol B1-treated AMD cells; Bar 3—resveratrol B2-treated AMD cells; Bar 4—resveratrol B3-treated AMD cells; Bar 5—resveratrol B4-treated AMD cells; Bar 6—resveratrol B5-treated AMD cells; Bar 7—resveratrol B6-treated AMD cells. Data are presented as mean ± SEM; * *p* < 0.05; *** *p* < 0.001.

**Figure 12:**
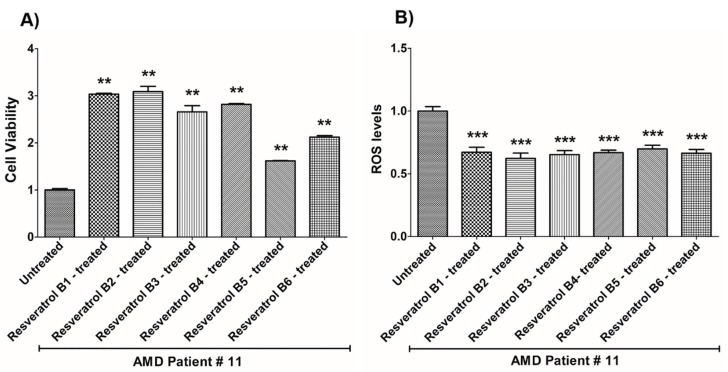
Effects of resveratrol formulations on cell viability (**A**) and ROS levels (**B**) in AMD patient #11. Bar 1—untreated AMD cells; Bar 2—resveratrol B1-treated AMD cells; Bar 3—resveratrol B2-treated AMD cells; Bar 4—resveratrol B3-treated AMD cells; Bar 5—resveratrol B4-treated AMD cells; Bar 6—resveratrol B5-treated AMD cells; Bar 7—resveratrol B6-treated AMD cells. Data are presented as mean ± SEM; ** *p* < 0.01; *** *p* < 0.001.

**Figure 13:**
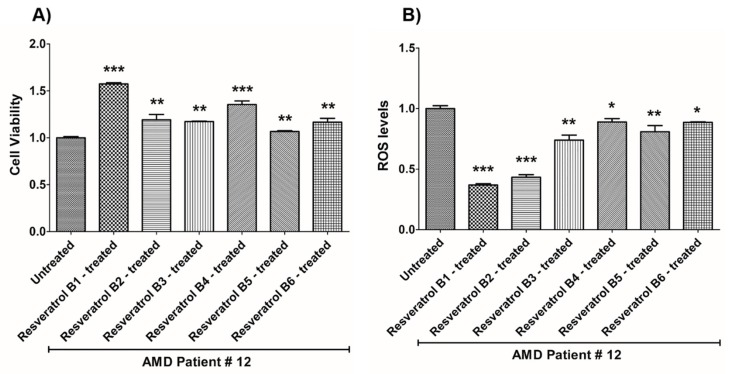
Effects of resveratrol formulations on cell viability (**A**) and ROS levels (**B**) in AMD patient #12. Bar 1—untreated AMD cells; Bar 2—resveratrol B1-treated AMD cells; Bar 3—resveratrol B2-treated AMD cells; Bar 4—resveratrol B3-treated AMD cells; Bar 5—resveratrol B4-treated AMD cells; Bar 6—resveratrol B5-treated AMD cells; Bar 7—resveratrol B6-treated AMD cells. Data are presented as mean ± SEM; * *p* < 0.05; ** *p* < 0.01; *** *p* < 0.001.

**Figure 14:**
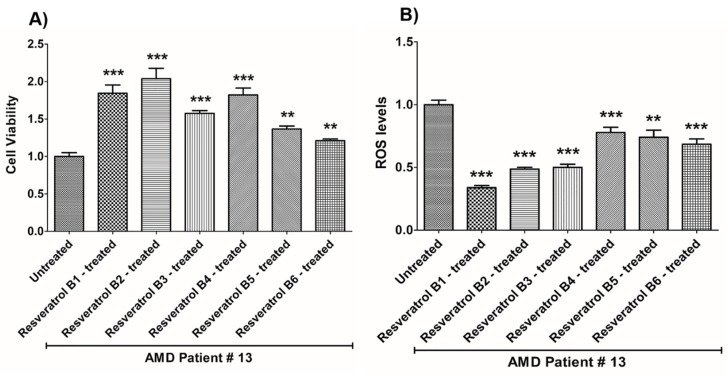
Effects of resveratrol formulations on cell viability (**A**) and ROS levels (**B**) in AMD patient #13. Bar 1—untreated AMD cells; Bar 2—resveratrol B1-treated AMD cells; Bar 3—resveratrol B2-treated AMD cells; Bar 4—resveratrol B3-treated AMD cells; Bar 5—resveratrol B4-treated AMD cells; Bar 6—resveratrol B5-treated AMD cells; Bar 7—resveratrol B6-treated AMD cells. Data are presented as mean ± SEM; ** *p* < 0.01, *** *p* < 0.001.

In contrast, on average in the AMD cybrids, the resveratrol B1 increased cell viability by 56.65%. Resveratrol Brand 1 (B1)-treated AMD RPE cells had significantly improved cell viability compared to their untreated counterparts (UN) in all AMD cybrids: AMD Patient #1 cybrid–41.5% increase, *p* = 0.0025 (Figure 2A, Table 3a); AMD Patient #2 cybrid–25.5% increase; *p* = 0.0159 (Figure 3A, Table 4a); AMD Patient #3 cybrid–31% increase; *p* = 0.0003 (Figure 4A, Table 5a); AMD Patient #4 cybrid–25.1% increase; *p* = 0.0294 (Figure 5A, Table 6a); AMD Patient #5 cybrid–42% increase; *p* = 0.0021 (Figure 6A, Table 7a); AMD Patient #6 cybrid–21.8% increase; *p* = 0.0139 (Figure 7A, Table 8a); AMD Patient #7 cybrid–59.6% increase; *p* = 0.0002 (Figure 8A, Table 9a); AMD Patient #8 cybrid–33% increase; *p* = 0.0050 (Figure 9A, Table 10a); AMD Patient #9 cybrid–61.1% increase; *p* = 0.0025 (Figure 10A, Table 11a); AMD Patient #10 cybrid–50.9% increase; *p* = 0.0002 (Figure 11A, Table 12a); AMD Patient #11 cybrid–203.4% increase; *p* = 0.0034 (Figure 12A, Table 13a); AMD Patient #12 cybrid–57.3% increase; *p* = 0.0005 (Figure 13A, Table 14a); AMD Patient #13 cybrid–84.3% increase; *p* = 0.0002 (Figure 14A, Table 15a).

#### 3.1.2. ROS Levels

Treatment with resveratrol Brand 1 (B1) caused significant reduction in NL RPE cybrid cells (49.15% decrease, *p* = 0.0006) (Figure 1B, Table 2b). Wildtype ARPE-19 cells treated with resveratrol Brand 1 showed lower ROS levels compared to their untreated counterparts (51.76% decrease; *p* = 0.0006) (Figure 1D, Table 2d).

Moreover, resveratrol Brand 1 (B1)-treated AMD RPE cybrid cells had significantly reduced ROS levels (48.75%) compared to their untreated counterparts in all AMD patients: AMD Patient #1 cybrid—37.98% decrease, *p* = 0.0012 (Figure 2B, Table 3b); AMD Patient #2 cybrid—56.1% decrease; *p* = 0.0002 (Figure 3B, Table 4b); AMD Patient #3 cybrid—66.68% decrease, *p* = 0.0014 (Figure 4B, Table 5b); AMD Patient #4 cybrid—55.75% decrease; *p* = 0.0043 (Figure 5B, Table 6b); AMD Patient #5 cybrid—40.36% decrease; *p* = 0.0012 (Figure 6B, Table 7b); AMD Patient #6 cybrid—46.93% decrease; *p* = 0.0002 (Figure 7B, Table 8b); AMD Patient #7 cybrid—41.1% decrease; *p* = 0.0002 (Figure 8B, Table 9b); AMD Patient #8 cybrid—69.13% decrease; *p* = 0.0003 (Figure 9B, Table 10b); AMD Patient #9 cybrid—18.01% decrease; *p* = 0.0242 (Figure 10B, Table 11b); AMD Patient #10 cybrid—39.69% decrease; *p* = 0.0006 (Figure 11B, Table 12b); AMD Patient #11 cybrid—32.84% decrease; *p* = 0.0002 (Figure 12B, Table 13b); AMD Patient #12 cybrid—63.04% decrease; *p* = 0.0001 (Figure 13B, Table 14b); AMD Patient #13 cybrid—66.14% decrease; *p* = 0.0007 (Figure 14B, Table 15b).

### 3.2. Effects of Resveratrol Brand 2 (B2)

#### 3.2.1. Cell Viability

Treatment with resveratrol B2 i.e., capsules containing 250 mg of *trans*-resveratrol, led to higher cell viability in normal (NL) cybrid cells (23.2% increase, *p* = 0.0175) (Figure 1A, Table 1a) and in wildtype ARPE-19 cells (40% increase, *p* = 0.0012) (Figure 1C, Table 2c).

On average, resveratrol Brand 2 (B2)-treated AMD RPE cells had significantly improved cell viability (56.57%) compared to their untreated AMD cybrids: AMD Patient #1 cybrid—52.2% increase, *p* = 0.0025; (Figure 2A, Table 3a); AMD Patient #2 cybrid—39% increase; *p* = 0.0159; (Figure 3A, Table 4a); AMD Patient #3 cybrid—48% increase; *p* = 0.0014; (Figure 4A, Table 5a); AMD Patient #4 cybrid—34.3% increase; *p* = 0.0139; (Figure 5A, Table 6a); AMD Patient #5 cybrid—35.2% increase; *p* = 0.0021; (Figure 6A, Table 7a); AMD Patient #6 cybrid—11.3% increase; *p* = 0.0498; (Figure 7A, Table 8a); AMD Patient #7 cybrid—47.5% increase; *p* = 0.0002; (Figure 8A, Table 9a); AMD Patient #8 cybrid—28.6% increase; p=0.0286; (Figure 9A, Table 10a); AMD Patient #9 cybrid—63% increase; *p* = 0.0025; (Figure 10A, Table 11a); AMD Patient #10 cybrid—44.4% increase; *p* = 0.0002; (Figure 11A, Table 12a); AMD Patient #11 cybrid—209% increase; *p* = 0.0034; (Figure 12A, Table 13a); AMD Patient #12 cybrid—19.1% increase; *p* = 0.0043; (Figure 13A, Table 14a); AMD Patient #13 cybrid—103.8% increase; *p* = 0.0002; (Figure 14A, Table 15a).

#### 3.2.2. ROS Levels

Treatment with resveratrol Brand 2 (B2) caused significant reduction in NL RPE cybrid cells (41.71% decrease, *p* = 0.0006) (Figure 1B, Table 2b). ARPE-19 cells treated with resveratrol Brand 2 showed lower ROS levels compared to their untreated counterparts (50.17% decrease; *p* = 0.0006) (Figure 1D, Table 2d).

Furthermore, resveratrol Brand 2 (B2)-treated AMD RPE cells had significantly reduced ROS levels (46.82%) compared to their untreated counterparts in all AMD patients: AMD Patient #1 cybrid—38.43% decrease, *p* = 0.0012; (Figure 2B, Table 3b); AMD Patient #2 cybrid—59.08% decrease; *p* = 0.0002; (Figure 3B, Table 4b); AMD Patient #3 cybrid—49.79% decrease; *p* = 0.0014; (Figure 4B, Table 5b); AMD Patient #4 cybrid—56.32% decrease; *p* = 0.0043; (Figure 5B, Table 6b); AMD Patient #5 cybrid—43.83% decrease; *p* = 0.0012; (Figure 6B, Table 7b); AMD Patient #6—47.11% decrease; *p* = 0.0002; (Figure 7B, Table 8b); AMD Patient #7 cybrid—42.84% decrease; *p* = 0.0002; (Figure 8B, Table 9b); AMD Patient #8 cybrid—63.83% decrease; *p* = 0.0003; (Figure 9B, Table 10b); AMD Patient #9 cybrid—24.31% decrease; *p* = 0.0061; (Figure 10B, Table 11b); AMD Patient #10 cybrid—37.53% decrease; *p* = 0.0006; (Figure 11B, Table 12b); AMD Patient #11 cybrid—37.66% decrease; *p* = 0.0002; (Figure 12B, Table 13b); AMD Patient #12 cybrid—56.68% decrease; *p* = 0.0009; (Figure 13B, Table 14b); AMD Patient #13 cybrid—51.29% decrease; *p* = 0.0007; (Figure 14B, Table 15b).

### 3.3. Effects of Resveratrol Brand 3 (B3)

#### 3.3.1. Cell Viability

Treatment with resveratrol B3 i.e., capsules containing 100 mg *trans*-resveratrol, led to significantly higher cell viability in normal (NL) cybrid cells (35.1% increase, *p* = 0.0041) (Figure 1A, Table 2a) and but not in Wildtype ARPE-19 cells (8.4% increase, *p* = 0.2619 (ns)) (Figure 1C, Table 2c).

Resveratrol Brand 3 (B3)-treated AMD RPE cells had significantly improved cell viability (40.92%) compared to their untreated AMD cybrids: AMD Patient #1 cybrid—32.8% increase, *p* = 0.0025; (Figure 2A, Table 3a); AMD Patient #2 cybrid—9.2% increase; *p* = 0.0357; (Figure 3A, Table 4a); AMD Patient #3 cybrid—57.9% increase; *p* = 0.0014; (Figure 4A, Table 5a); AMD Patient #4 cybrid—18.1% increase; *p* = 0.0498; (Figure 5A, Table 6a); AMD Patient #5 cybrid—23.9% increase; *p* = 0.0021; (Figure 6A, Table 7a); AMD Patient #6 cybrid—16.9% increase; *p* = 0.0294; (Figure 7A, Table 8a); AMD Patient #7 cybrid—35.5% increase; *p* = 0.0003; (Figure 8A, Table 9a); AMD Patient #8 cybrid—27.7% increase; *p* = 0.0095; (Figure 9A, Table 10a); AMD Patient #9 cybrid—37.8% increase; *p* = 0.0025; (Figure 10A, Table 11a); AMD Patient #10 cybrid—31.4% increase; *p* = 0.0002; (Figure 11A, Table 12a); AMD Patient #11 cybrid—165.9% increase; *p* = 0.0034; (Figure 12A, Table 13a); AMD Patient #12 cybrid—17.3% increase; *p* = 0.0095; (Figure 13A, Table 14a); AMD Patient #13 cybrid—57.6% increase; *p* = 0.0002; (Figure 14A, Table 15a).

#### 3.3.2. ROS Levels

Treatment with resveratrol Brand 3 (B3) caused significant reduction in NL RPE cells (44.19% decrease, *p* = 0.0006) (Figure 1B, Table 2b). ARPE-19 cells treated with resveratrol Brand 3 showed lower ROS levels compared to their untreated counterparts (49.45% decrease; *p* = 0.0006) (Figure 1D, Table 2d).

On average, the resveratrol Brand 3 (B3)-treated AMD RPE cells had significantly reduced ROS levels (38.5%) compared to their untreated counterparts in all AMD cybrids: AMD Patient #1 cybrid—35.83% decrease, *p* = 0.0022; (Figure 2B, Table 3b); AMD Patient #2 cybrid—45.86% decrease; *p* = 0.0002; (Figure 3B, Table 4b); AMD Patient #3 cybrid—54.96% decrease; *p* = 0.0014; (Figure 4B, Table 5b); AMD Patient #4 cybrid—50.5% decrease; *p* = 0.0079; (Figure 5B, Table 6b); AMD Patient #5 cybrid—32.1% decrease; *p* = 0.0022; (Figure 6B, Table 7b); AMD Patient #6 cybrid—41.44% decrease; *p* = 0.0002; (Figure 7B, Table 8b); AMD Patient #7 cybrid—36.48% decrease; *p* = 0.0006; (Figure 8B, Table 9b); AMD Patient #8 cybrid—44.76% decrease; *p* = 0.0003; (Figure 9B, Table 10b); AMD Patient #9 cybrid—17.15% decrease; *p* = 0.0095; (Figure 10B, Table 11b); AMD Patient #10 cybrid—30.69% decrease; *p* = 0.0006; (Figure 11B, Table 12b); AMD Patient #11 cybrid—34.71% decrease; *p* = 0.0002; (Figure 12B, Table 13b); AMD Patient #12 cybrid—26.04% decrease; *p* = 0.0013; (Figure 13B, Table 14b); AMD Patient #13 cybrid—49.99% decrease; *p* = 0.0007; (Figure 14B, Table 15b).

### 3.4. Effects of Resveratrol Brand 4 (B4)

#### 3.4.1. Cell Viability

Treatment with resveratrol B4 i.e., capsules containing 500 mg red wine complex and an unspecified amount of *trans*-resveratrol, led to higher cell viability in normal (NL) cybrid cells (50.4% increase, *p* = 0.0006) (Figure 1A, Table 2a) and in wildtype ARPE-19 cells (66.9% increase, *p* = 0.0012) (Figure 1C, Table 2c).

On average, resveratrol Brand 4 (B4)-treated AMD RPE cells had significantly improved cell viability (66.9%) compared to their untreated counterparts in all patients: AMD Patient #1 cybrid—73.9% increase, *p* = 0.0025; (Figure 2A, Table 3a); AMD Patient #2 cybrid—28% increase; *p* = 0.0016; (Figure 3A, Table 4a); AMD Patient #3 cybrid—53.4% increase; *p* = 0.0014; (Figure 4A, Table 5a); AMD Patient #4 cybrid—39.8% increase; *p* = 0.0084; (Figure 5A, Table 6a); AMD Patient #5 cybrid—67% increase; *p* = 0.0021; (Figure 6A, Table 7a); AMD Patient #6 cybrid—47.9% increase; *p* = 0.0084; (Figure 7A, Table 8a); AMD Patient #7 cybrid—75.6% increase; *p* = 0.0003; (Figure 8A, Table 9a); AMD Patient #8 cybrid—38.2% increase; *p* = 0.0040; (Figure 9A, Table 10a); AMD Patient #9 cybrid—70.2% increase; *p* = 0.0025; (Figure 10A, Table 11a); AMD Patient #10 cybrid—76.8% increase; *p* = 0.0002; (Figure 11A, Table 12a); AMD Patient #11 cybrid—181.9% increase; *p* = 0.0034; (Figure 12A, Table 13a); AMD Patient #12 cybrid—35.4% increase; *p* = 0.0007; (Figure 13A, Table 14a); AMD Patient #13 cybrid—82.1% increase; *p* = 0.0002; (Figure 14A, Table 15a).

#### 3.4.2. ROS Levels

Treatment with resveratrol Brand 4 (B4) caused significant reduction in NL RPE cybrid cells (34.52% decrease, *p* = 0.0006) (Figure 1B, Table 2b). Wildtype ARPE-19 cells treated with resveratrol Brand 4 showed lower ROS levels compared to their untreated counterparts (27.38% decrease; *p* = 0.0023) (Figure 1D, Table 2d).

On average, resveratrol Brand 4 (B4)-treated AMD RPE cells had significantly reduced ROS levels (16.91%) compared to their untreated counterparts in all AMD cybrids: AMD Patient #1 cybrid—22.68% decrease, *p* = 0.0022; (Figure 2B, Table 3b); AMD Patient #2 cybrid—18.26% decrease; *p* = 0.0040; (Figure 3B, Table 4b); AMD Patient #3 cybrid—19.68% decrease; *p* = 0.0021; (Figure 4B, Table 5b); AMD Patient #4 cybrid—14.99% decrease; *p* = 0.0714; (Figure 5B, Table 6b); AMD Patient #5 cybrid—*p* = 1.0 (non-significant); (Figure 6B, Table 7b); AMD Patient #6 cybrid—21.94% decrease; *p* = 0.0062; (Figure 7B, Table 8b); AMD Patient #7 cybrid—25.23% decrease; *p* = 0.0289; (Figure 8B, Table 9b) AMD Patient #8 cybrid—17.06% decrease; *p* = 0.0047; (Figure 9B, Table 10b); AMD Patient #9 cybrid—13.57% decrease; *p* = 0.4000; (Figure 10B, Table 11b); AMD Patient #10 cybrid—*p* = 0.2667; (Figure 11B, Table 12b); AMD Patient #11 cybrid—33.18% decrease; *p* = 0.0002; (Figure 12B, Table 13b); AMD Patient #12 cybrid—11.05% decrease; *p* = 0.0100; (Figure 13B, Table 14b); AMD Patient #13 cybrid—22.14% decrease; *p* = 0.0007; (Figure 14B, Table 15b).

### 3.5. Effects of Resveratrol Brand 5 (B5)

#### 3.5.1. Cell Viability

Treatment with resveratrol B5 i.e., resveratrol from Polygonum cuspidatum root and containing an unspecified amount of *trans*-resveratrol, resulted in higher cell viability in normal (NL) cybrid (15.9% increase, *p* = 0.0350) (Figure 1A, Table 2a) and in ARPE-19 cells (44.2% increase, *p* = 0.0012) (Figure 1C, Table 2c).

On average, resveratrol Brand 5 (B5)-treated AMD RPE cells had significantly improved cell viability (29.1%) compared to their untreated counterparts in all patients: AMD Patient #1 cybrid—22.3% increase, *p* = 0.0079; (Figure 2A, Table 3a); AMD Patient #2 cybrid—25% increase; *p* = 0.0025; (Figure 3A, Table 4a); AMD Patient #3 cybrid—42.7% increase; *p* = 0.0014; (Figure 4A, Table 5a); AMD Patient #4 cybrid—14.8% increase; *p* = 0.0195; (Figure 5A, Table 6a); AMD Patient #5 cybrid—23% increase; *p* = 0.0021; (Figure 6A, Table 7a); AMD Patient #6 cybrid—53.4% increase; *p* = 0.0084; (Figure 7A, Table 8a); AMD Patient #7 cybrid—17.7% increase; *p* = 0.0003; (Figure 8A, Table 9a); AMD Patient #8 cybrid—26.5% increase; *p* = 0.0095; (Figure 9A, Table 10a); AMD Patient #9 cybrid—21.9% increase; *p* = 0.0025; (Figure 10A, Table 11a); AMD Patient #10 cybrid—32.7% increase; *p* = 0.0002; (Figure 11A, Table 12a); AMD Patient #11 cybrid—61.8% increase; *p* = 0.0034; (Figure 12A, Table 13a); AMD Patient #12 cybrid—*p* = 0.0095 (non‑significant); (Figure 13A, Table 14a); AMD Patient #13 cybrid—36.6% increase; *p* = 0.0012; (Figure 14A, Table 15a).

#### 3.5.2. ROS Levels

Treatment with resveratrol Brand 5 (B5) caused significant reduction in NL RPE cybrid cells (33.98% decrease, *p* = 0.0006) (Figure 1B, Table 2b). Wildtype ARPE-19 cells treated with resveratrol Brand 5 showed lower ROS levels compared to their untreated counterparts (28.67% decrease; *p* = 0.0006) (Figure 1D, Table 2d).

Resveratrol Brand 5 (B5)-treated AMD RPE cells had significantly reduced ROS levels (22%) compared to their untreated counterparts in all AMD cybrid cells lines: AMD Patient #1 cybrid—31.93% decrease, *p* = 0.0022; (Figure 2B, Table 3b); AMD Patient #2 cybrid—24.81% decrease; *p* = 0.0002; (Figure 3B, Table 4b); AMD Patient #3 cybrid—*p* = 0.5074; (Figure 4B, Table 5b); AMD Patient #4 cybrid—36.55% decrease; *p* = 0.0079; (Figure 5B, Table 6b); AMD Patient #5 cybrid—20.35% decrease; *p* = 0.0238; (Figure 6B, Table 7b); AMD Patient #6 cybrid—24.91% decrease; *p* = 0.0007; (Figure 7B, Table 8b); AMD Patient #7 cybrid—22.02% decrease; *p* = 0.0379; (Figure 8B, Table 9b); AMD Patient #8 cybrid—13.5% decrease; *p* = 0.0303; (Figure 9B, Table 10b); AMD Patient #9 cybrid—*p* = 0.4000 (non‑significant); (Figure 10B, Table 11b); AMD Patient #10 cybrid—24.92% decrease; *p* = 0.0006; (Figure 11B, Table 12b); AMD Patient #11 cybrid—30.16% decrease; *p* = 0.0002; (Figure 12B, Table 13b); AMD Patient #12 cybrid—19.12% decrease; *p* = 0.0019; (Figure 13B, Table 14b); AMD Patient #13 cybrid—25.98% decrease; *p* = 0.0043; (Figure 14B, Table 15b).

### 3.6. Effects of Resveratrol Brand 6 (B6)

#### 3.6.1. Cell Viability

Treatment with resveratrol B6 i.e., resveratrol from *Polygonum cuspidatum* root and containing an unspecified amount of *trans*-resveratrol, resulted in higher cell viability in normal (NL) cybrid cells (19.8% increase, *p* = 0.0221) (Figure 1A, Table 2a) and in wildtype ARPE-19 cells (25.9% increase, *p* = 0.0023) (Figure 1C, Table 2c).

On average, resveratrol Brand 6 (B6)-treated AMD RPE cells had significantly improved cell viability (41.45%) compared to their untreated counterparts in all patients: AMD Patient #1 cybrid—46.7% increase, *p* = 0.0025; (Figure 2A, Table 3a); AMD Patient #2 cybrid—24.1% increase; *p* = 0.0016; (Figure 3A, Table 3a); AMD Patient #3 cybrid—16.9% increase; *p* = 0.0175; (Figure 4A, Table 5a); AMD Patient #4 cybrid—47.5% increase; *p* = 0.0084; (Figure 5A, Table 6a); AMD Patient #5 cybrid—50.2% increase; *p* = 0.0021; (Figure 6A, Table 7a); AMD Patient #6 cybrid—47.4% increase; *p* = 0.0106; (Figure 7A, Table 8a); AMD Patient #7 cybrid—48.8% increase; *p* = 0.0003; (Figure 8A, Table 9a); AMD Patient #8 cybrid—*p* = 0.8000 (non-significant); (Figure 9A, Table 10a); AMD Patient #9 cybrid—55.9% increase; *p* = 0.0025; (Figure 10A, Table 11a); AMD Patient #10 cybrid—53% increase; *p* = 0.0002; (Figure 11A, Table 12a); AMD Patient #11 cybrid—112.2% increase; *p* = 0.0034; (Figure 12A, Table 13a); AMD Patient #12 cybrid—16.6% increase; *p* = 0.0027; (Figure 13A, Table 14a); AMD Patient #13 cybrid—21% increase; *p* = 0.0093; (Figure 14A, Table 15a).

#### 3.6.2. ROS Levels

Treatment with resveratrol Brand 6 (B6) caused significant reduction in NL RPE cells (32.55% decrease, *p* = 0.0006) (Figure 1B, Table 2b). ARPE-19 cells treated with resveratrol Brand 6 showed lower ROS levels compared to their untreated counterparts (31.11% decrease; *p* = 0.0012) (Figure 1D, Table 2d).

On average, resveratrol Brand 6 (B6)-treated AMD RPE cells had significantly reduced ROS levels (23%) compared to their untreated counterparts in all AMD cybrid cell lines: AMD Patient #1 cybrid—30.79% decrease, *p* = 0.0022; (Figure 2B, Table 3b); AMD Patient #2 cybrid—21.63% decrease; *p* = 0.0040; (Figure 3B, Table 4b); AMD Patient #3 cybrid—17.2% decrease; *p* = 0.0294; (Figure 4B, Table 5b); AMD Patient #4 cybrid—26.44% decrease; *p* = 0.0079; (Figure 5B, Table 6b); AMD Patient #5 cybrid—16.39% decrease; *p* = 0.0238; (Figure 6B, Table 7b); AMD Patient #6 cybrid—19.4% decrease; *p* = 0.0109; (Figure 7B, Table 8b); AMD Patient #7 cybrid—31.11% decrease; *p* = 0.0019; (Figure 8B, Table 9b); AMD Patient #8 cybrid—28.33% decrease; *p* = 0.0006; (Figure 9B, Table 10b); AMD Patient #9 cybrid—18.38% decrease; *p* = 0.0159; (Figure 10B, Table 11b); AMD Patient #10 cybrid—13.47% decrease; *p* = 0.0262; (Figure 11B, Table 12b); AMD Patient #11 cybrid—33.61% decrease; *p* = 0.0002; (Figure 12B, Table 13b); AMD Patient #12 cybrid—11.33% decrease; *p* = 0.0317; (Figure 13B, Table 14b); AMD Patient #13 cybrid—31.56% decrease; *p* = 0.0007; (Figure 14B, Table 15b).

Table 16a,b summarize the effects of treatment with various resveratrol formulations in AMD patients.

## 4. Discussion

The current study demonstrates the biological effects of resveratrol in AMD RPE transmitochondrial cells in which the mitochondria are derived from AMD patients and the nuclei come from Rho0 ARPE-19 cell lines. Cybrid status and that the cybrids have acquired their mtDNAs from the donor individuals was confirmed using allelic discrimination, Sanger sequencing, and next-generation sequencing. In our recent work [8,9,10,11], the ‘mtDNA damage’ that the AMD RPE cybrid cells implicitly carry from the AMD patients has been extensively characterized and various endpoints measured in terms of mitochondrial and cellular health have revealed significant differences including epigenetic alterations, downregulation of mitochondrial transcription and replication genes, mtDNA fragmentation, and reduction in mtDNA copy numbers observed in AMD RPE cybrid cells compared to normal RPE cybrid cells. We found that OTC resveratrol dietary supplements protect AMD RPE transmitochondrial cells against cell death and oxidative stress in vitro, indicating a potential role of resveratrol as a nutritional therapeutic candidate in AMD disease.

Since RPE cell death and oxidative stress are characteristic of AMD disease pathology, in this in vitro study, we sought to compare six different resveratrol brands (with different manufacturers) with regard to their potential in preventing cell death and reducing reactive oxygen species (ROS) in AMD RPE transmitochondrial cell lines. In the transmitochondrial AMD ARPE-19 cybrid cells, since the nuclear content derived from ARPE-19 cells is the same and the cells differ only in the mitochondrial DNA content, which is derived from AMD patients, the idea is that the observed cellular and molecular changes could be attributed to variations in mitochondrial DNA that was obtained from AMD patients. This allows us to examine the mechanisms of retrograde signaling within the cybrid cells and provides an efficient model for in vitro screening of potential therapeutic candidates for AMD. All AMD patients used in this study have been clinically characterized, and genetic and clinical information of all patients is available as partly mentioned in Table 1. Morphological and functional evaluation of these AMD RPE transmitochondrial cell lines in our previous studies revealed significant mitochondrial and cellular damage as evidenced by apoptotic cell death, higher oxidative stress, low antioxidant content, lower numbers of mitochondria, and higher mtDNA fragmentation in AMD RPE cells [9,10,11,12,13]. Therefore, AMD RPE transmitochondrial cell lines serve as a good in vitro model to test the effects of resveratrol as a potential over-the-counter candidate for AMD therapy. We tested a wide range of concentrations and chose 1000 µM as the final optimal working concentration for all experiments since it showed the maximum positive effects in pilot experiments. Although all six resveratrol formulations used here produced significant cell rescue effects in AMD cells, the degrees of positive effects differed with each patient. These inter-patient differences could be attributed to demographics with respect to age, type of AMD, and gender. The average age of the AMD patients used in his study was 79.7 ± 1.7 (Mean ± SEM) years. Of the 13 patients, there were eight wet AMD patients and five dry AMD patients; four females and nine males.

We examined cell viability of AMD RPE cells in response to resveratrol administration using the yellow tetrazolium MTT (3-(4,5-dimethylthiazolyl-2)-2,5-diphenyltetrazolium bromide) reagent, which is reduced by metabolically active live cells, in part by the action of dehydrogenase enzymes, to generate reducing equivalents such as NADH and NADPH. The resulting intracellular purple formazan is solubilized by DMSO and quantified by spectrophotometry. The chemiluminescence signal produced is proportional to the number of live cells, thereby allowing quantification of changes in the rate of cell viability. We found that resveratrol Brand 1, which was obtained as a ≥99% HPLC-purified *trans*‑resveratrol powder, showed an average increase in cell viability i.e., 56.65% in AMD patient cybrid cell lines compared to their untreated counterparts. Brand 1 was relatively consistent in its potential to increase cell viability in the AMD patient cell lines. This consistency and efficiency of Brand 1 probably could be attributed to its high content of *trans*-resveratrol, which is the predominant bioactive form of resveratrol and is known to exhibit a broad spectrum of pharmacological properties including antioxidant, anti-cancer, anti-mutagenic, neuroprotective, cardioprotective, anti‑inflammatory, and anti-aging activities [14]. In addition to 250 mg of *trans*‑resveratrol, Brand 2 also contained 100 mg of Quercetin (as Quercetin dihydrate), which is a polyphenolic flavonoid commonly found in vegetables and fruits. Quercetin is reported to have a variety of health benefits such as attenuation of anaphylactic reactions, anti-inflammatory, antioxidant, neuroprotective, and anti-cancer activities [15]. This is probably why treatment with resveratrol Brand 2 showed an average increase in cell viability of 56.57%, which was similar to that observed with Brand 1 purified formulation. Brand 3, which provides 100 mg *trans*‑resveratrol from *Polygonum cuspidatum* root extract and grape (*Vitis vinifera*) seed and skin extract, showed an average higher cell viability of 40.92% in AMD cells. Brand 4 which contained unspecified amounts of *trans*-resveratrol from *Polygonum cuspidatum* root, grape seed, and red wine extract, showed relatively higher average cell viability i.e., 66.9%, among the 13 AMD patient cybrid cell lines studied. Treatment with Brand 6 resveratrol, which was derived from *Polygonum cuspidatum* root, led to an average increase in viable cell numbers by 41.56%. The manufacturers of Brand 6 did not specify the amount, if any, of *trans*-resveratrol this formulation contains. Brand 6 resveratrol did not produce any significant increase in live cell number in AMD cells derived from dry AMD patient #8. However, based on our data, one can assume its *trans*-resveratrol content was similar to those in other formulations. Brand 5 had an average cell viability increase of 29%. Therefore, compared to other formulations, the cell rescue action of Brand 5 was reduced in AMD patient cell lines and no significant effect was observed in ROS levels in AMD cells derived from the wet AMD patients #3 and #9. This may have been because of lower *trans*‑resveratrol content in Brand 5 formulation. Although Brand 5 resveratrol was derived from *Polygonum cuspidatum* root, no information regarding its *trans*‑resveratrol content, if any, was provided. Of all the patients’ cybrid cell lines, resveratrol showed the maximum cell viability increase in AMD cells derived from wet AMD patient #11. The Patient #11 cybrid had more original damage, but they were rescued the most with all of the resveratrol. The untreated cybrid cells had extraordinarily lower cell viability to begin with, but resveratrol improved the cell viability substantially, ranging from 0.75-fold to 2-fold higher than untreated.

These positive effects of resveratrol formulations on cell viability are consistent with previous studies, which have established the role of resveratrol as an anti-apoptotic agent. For instance, Seong et al. demonstrated that resveratrol protects against in vivo ischemia-induced retinal injury by regulating the apoptotic caspase pathway. Resveratrol prevents retinal cell death and mitigates the deleterious effects of retinal injury via downregulation of *Caspase-3* and *Caspase-8* mRNA transcripts and protein [16]. Resveratrol plays a neuroprotective role in the retina by preventing retinal ganglion cell (RGC) loss via inhibition of the BAX-Caspase-3-dependent apoptotic pathway, attenuation of trauma-induced reactive gliosis, and decreasing the levels of pro‑inflammatory cytokines [17]. Lindsey et al. showed that treatment with resveratrol after optic nerve injury protected RGC dendrites by modulating the UPR (unfolded protein response) proteins namely BiP, CHOP, and XBP-1 [18]. Resveratrol alleviates hypoxia-induced apoptosis in retinas in vivo by downregulating *Caspase-3* and *Caspase‑9* genes [19]. Resveratrol’s mechanism of cytoprotection varies with the type of insult: Resveratrol exerts its effects by inhibition of caspase activity to mitigate the chemically induced oxidative stress damage; resveratrol regulates tau phosphorylation at Ser422 in response to DNA damage [20].

As an indicator of ROS levels in cells, we used the cell-permeant 2′,7′-dichlorodihydrofluorescein diacetate (H2DCFDA), which is a chemically reduced form of fluorescein. Upon cleavage of the acetate groups by intracellular esterases and oxidation, the nonfluorescent H2DCFDA is converted to the highly fluorescent 2′,7′-dichlorofluorescein (DCF), the intensity of which is directly proportional to ROS levels in cells [21].

In this study, we tested the effects of resveratrol on ROS levels because mitochondrial ROS production underlies oxidative damage and is crucial in retrograde redox signaling. Intracellular ROS are produced as natural byproducts of oxygen metabolism within organelles such as mitochondria, endoplasmic reticulum, and peroxisomes. In the mitochondria, ROS are generated during ATP formation in oxidative phosphorylation [22]. The free radical theory of aging by Denham Harman attributes aging to free radical accumulation over time [23]. Mitochondrial theory of aging implicates the mitochondria as the primary site of both ROS generation as well as ROS-induced radical damage [24]. Superoxide anion and hydrogen peroxide constitute mitochondrial ROS, and hypoxia, apoptosis, and p53 activation are some of the stimuli that induce mitochondrial ROS production. Several external agents such as pollutants, tobacco, smoke, drugs, xenobiotics, or radiation may act as exogenous stimuli. The deleterious effects of ROS include, but are not limited to, damage to DNA and/or RNA, amino acid oxidation, and lipid peroxidation [25]. To counteract the toxic effects of ROS, the endogenous antioxidant systems come into play—these include superoxide dismutase, catalase, glutathione peroxidase, coenzyme Q, Uric acid, plasma bilirubin, dihydrolipoic acid, and metallothionein. However, diminished antioxidant levels with aging and excessive ROS generation causes redox imbalance resulting in oxidative stress, which is reportedly associated with the pathogenesis of many age-related diseases including AMD [26]. Our recent study has shown elevated mitochondrial superoxide and reduced antioxidant levels in AMD RPE cells [8].

Resveratrol is known to scavenge free radicals, quench ROS, and upregulate endogenous antioxidants such as superoxide dismutase and catalase [27]. In the present study, the relatively high *trans*-resveratrol content of Brand 1 and Brand 2 may have accounted for those being the most effective formulations in lowering ROS levels by 48.75% and 46.82%, respectively. in AMD patient cell lines, suggesting their potential to decrease oxidative stress. Brand 3 was the third best resveratrol formulation regarding its ROS lowering potential, causing a 38.5% reduction in ROS levels. Brands 4, -5, and -6 reduced ROS levels by 16.9%, 22%, and 23%, respectively, suggesting their significant but comparatively reduced potential to scavenge ROS in AMD cells. Since the amount of *trans*-resveratrol in the last three brands was unknown, their lower ROS quenching activity may be attributable to relatively lower *trans*-resveratrol content. The possible mechanism of action of *trans*-resveratrol’s cytoprotective action is via activation of Sirtuins, which in turn promote longevity in cells thereby delaying aging [15]. Our results are corroborated by previous findings. For example, Xin et al. demonstrated that resveratrol reduces hypoxic stress by decreasing hypoxia-induced upregulation of *HIF-1* (*Hypoxia-Inducible Factor-1*), *Trx1* (*Thioredoxin 1*), and *Trx2* (*Thioredoxin 2*) transcripts in rat retinas [19]. Notably, treatment with resveratrol counteracts ROS-induced cellular injury by inducing various cytoprotective antioxidants and phase 2 enzymes including catalase, superoxide dismutase, glutathione, glutathione reductase, glutathione peroxidase, glutathione S-transferase (GST), and NAD(P)H:quinone oxidoreductase-1 (NOQ1) [28]. Resveratrol reduces hyperglycemia-induced oxidative stress damage by modulating SIRT1 deacetylase activity and the SIRT1/FOXO3a pathway [29].

Resveratrol formulations tested in the current study produced similar cytoprotective effects in wet and dry AMD patient cell lines. Since the AMD cell lines have damaged AMD mitochondria, we speculate resveratrol exerts its protective effects by improving mitochondrial health and function. This assumption is supported by recent studies that demonstrated that resveratrol exerts its anti-aging effects in zebrafish retina by enhancing mitochondrial quality and growth, suppressing Akt/mTOR pathway, and upregulating Ampk/Sirt1/PGC-1α [30]. Furthermore, systemic treatment in AMD patients that may have modulated the mitochondrial DNA status of patients could certainly influence the AMD cybrid cells’ response to resveratrol. However, at this time. we cannot confirm this mechanism.

Cytoprotective effects of resveratrol observed in our study support the results of clinical studies in which resveratrol administration was found to be safe. Clinical trials to test the safety and efficacy of resveratrol for Neovascular AMD were initiated in Europe in 2016. Clinical trials by Brown et al. have demonstrated that resveratrol intake is safe; clinical, biochemical, and hematologic evaluation revealed that resveratrol causes no serious adverse reactions during the study and follow-up visits. Although higher doses of resveratrol i.e., up to 5 mg, caused gastrointestinal discomfort, the symptoms were mild and of severity grade 1 [31]. Apart from safety, development of resveratrol therapeutic will require techniques that increase its bioavailability. In a Phase 1 randomized pilot study by Howells et al., micronized resveratrol was administrated to volunteers since micronization enables enhanced absorption, thereby increasing systemic availability. That study reported micronized resveratrol to have higher C_max_ i.e., maximum serum concentration than reported for equivalent dose of non-micronized resveratrol. Although further clinical investigations are required, the study does suggest micronization as a viable option for development of resveratrol formulation [32]. The Longevinex^®^ capsules which contain 100 mg of micronized and microencapsulated *trans*-resveratrol have been successfully used as a nutritional supplement for a long time.

As mentioned above, we used 1000 µM as the final optimal working concentration in this study since 1000 µM resveratrol produced the maximum positive effects in pilot experiments that tested a wide range of resveratrol concentrations in AMD RPE cells. However, additional studies are required to examine the potential of resveratrol at varying concentrations in the in vitro AMD RPE cell model. Furthermore, to shed light on its pharmacokinetics and distribution, further studies with resveratrol metabolites will be required since, upon absorption, resveratrol is rapidly metabolized to resveratrol sulfate and glucuronide conjugates and as dihydroresveratrol-sulfate and dihydroresveratrol-glucuronide.

In conclusion, although further validation, including in vitro studies and randomized, double-blinded, clinical trials, are required to validate the merit of resveratrol as an across the board ocular nutraceutical; our present study is novel as no previous study has examined the role of resveratrol in AMD RPE transmitochondrial cybrid cells, and our study establishes the role of over-the-counter resveratrol formulations in alleviating reactive oxygen species and improving cell viability in AMD transmitochondrial cell lines.

## Figures and Tables

**Table 1:** Age-related macular degeneration (AMD) patients’ and AMD retinal pigment epithelial (RPE) cell lines’ information.

AMD PATIENT #	GENDER	AGE	AMD TYPE
PATIENT #1	MALE	76	WET
PATIENT #2	FEMALE	75	WET
PATIENT #3	MALE	83	WET
PATIENT #4	MALE	74	WET
PATIENT #5	MALE	87	DRY
PATIENT #6	MALE	83	DRY
PATIENT #7	MALE	77	DRY
PATIENT #8	MALE	76	DRY
PATIENT #9	MALE	90	WET
PATIENT #10	FEMALE	84	WET
PATIENT #11	FEMALE	86	WET
PATIENT #12	MALE	69	DRY
PATIENT #13	FEMALE	76	WET

**Table 2:** Effects of resveratrol formulations on cell viability (**a**,**c**) and ROS levels (**b**,**d**) in normal cybrid cell lines (**a**,**b**) and ARPE-19 cell lines (**c**,**d**).

**(a) Normal Cybrid_Resveratrol Effects on Cell Viability.**			
**Cell Viability**	**Percent Increase/*p*-Value**	**Untreated** **Mean ± SEM**	**Resveratrol Brands** **Mean ± SEM**
NL UN vs. NL B1-treated	3.3% 0.7748 (ns)	1 ± 0.03821	1.033 ± 0.09471
NL UN vs. NL B2-treated	23.2% 0.0175	1 ± 0.03821	1.232 ± 0.06256
NL UN vs. NL B3-treated	35.1% 0.0041	1 ± 0.03821	1.351 ± 0.1249
NL UN vs. NL B4-treated	50.4% 0.0006	1 ± 0.03821	1.504 ± 0.08458
NL UN vs. NL B5-treated	15.9% 0.0350	1 ± 0.03821	1.159 ± 0.04787
NL UN vs. NL B6-treated	19.8% 0.0221	1 ± 0.03821	1.198 ± 0.05209
**(b) Normal Cybrid_Resveratrol Effects on ROS Levels.**			
**Cell Viability**	**Percent Decrease/*p*-Value**	**Untreated** **Mean ± SEM**	**Resveratrol Brands** **Mean ± SEM**
NL UN vs. NL B1-treated	49.15% 0.0006	1 ± 0.03147	0.5085 ± 0.02217
NL UN vs. NL B2-treated	41.71% 0.0006	1 ± 0.03147	0.5829 ± 0.02555
NL UN vs. NL B3-treated	44.19% 0.0006	1 ± 0.03147	0.5581 ± 0.01816
NL UN vs. NL B4-treated	34.52% 0.0006	1 ± 0.03147	0.6548 ± 0.02463
NL UN vs. NL B5-treated	33.98% 0.0006	1 ± 0.03147	0.6602 ± 0.01098
NL UN vs. NL B6-treated	32.55% 0.0006	1 ± 0.03147	0.6745 ± 0.03597
**(c) ARPE-19 cells Resveratrol Effects on Cell Viability.**			
**Cell Viability**	**Percent Increase/*p*-Value**	**Untreated** **Mean ± SEM**	**Resveratrol brands** **Mean ± SEM**
ARPE-19 UN vs. ARPE-19 B1-treated	3.5% 0.5476 (*n*s)	1 ± 0.03600	1.035 ± 0.02017
ARPE-19 UN vs. ARPE-19 B2-treated	40% 0.0012	1 ± 0.03600	1.400 ± 0.08388
ARPE-19 UN vs. ARPE-19 B3-treated	8.4% 0.2619 (*n*s)	1 ± 0.03600	1.084 ± 0.06225
ARPE-19 UN vs. ARPE-19 B4-treated	66.9% 0.0012	1 ± 0.03600	1.669 ± 0.1240 N = 7
ARPE-19 UN vs. ARPE-19 B5-treated	44.2% 0.0012	1 ± 0.03600	1.442 ± 0.03927
ARPE-19 UN vs. ARPE-19 B6-treated	25.9% 0.0023	1 ± 0.03600	1.259 ± 0.07003
**(d) ARPE-19 cells_Resveratrol Effects on ROS Levels.**			
**Cell Viability**	**Percent Decrease/*p*-Value**	**Untreated** **Mean ± SEM**	**Resveratrol Brands** **Mean ± SEM**
ARPE-19 UN vs. ARPE-19 B1-treated	51.76% 0.0006	1 ± 0.02965	0.4824 ± 0.01264
ARPE-19 UN vs. ARPE-19 B2-treated	50.17% 0.0006	1 ± 0.02965	0.4983 ± 0.02390
ARPE-19 UN vs. ARPE-19 B3-treated	49.45% 0.0006	1 ± 0.02965	0.5055 ± 0.01476
ARPE-19 UN vs. ARPE-19 B4-treated	27.38% 0.0023	1 ± 0.02965	0.7262 ± 0.03931
ARPE-19 UN vs. ARPE-19 B5-treated	28.67% 0.0006	1 ± 0.02965	0.7133 ± 0.01624
ARPE-19 UN vs. ARPE-19 B6-treated	31.11% 0.0012	1 ± 0.02965	0.6889 ± 0.02664

**Table 3:** Effects of resveratrol formulations on cell viability (**a**) and ROS levels (**b**) in AMD patient # 1.

**(a) AMD PATIENT #1_Resveratrol Effects on Cell Viability.**			
**Cell Viability**	**Percent Increase/*p*-Value**	**Untreated** **Mean ± SEM**	**Resveratrol Brands** **Mean ± SEM**
AMD UN vs. AMD B1-treated	41.5% 0.0025	1 ± 0.01234	1.415 ± 0.02189
AMD UN vs. AMD B2-treated	52.2% 0.0025	1 ± 0.01234	1.522 ± 0.1461
AMD UN vs. AMD B3-treated	32.8% 0.0025	1 ± 0.01234	1.328 ± 0.03817
AMD UN vs. AMD B4-treated	73.9% 0.0025	1 ± 0.01234	1.739 ± 0.06509
AMD UN vs. AMD B5-treated	22.3% 0.0079	1 ± 0.01234	1.223 ± 0.02813
AMD UN vs. AMD B6-treated	46.7% 0.0025	1 ± 0.01234	1.467 ± 0.01599
**(b) AMD PATIENT #1_Resveratrol Effects on ROS Levels.**			
**Ros Levels**	**Percent Decrease/*p*-Value**	**Untreated** **Mean ± SEM**	**Resveratrol Brands** **Mean ± SEM**
AMD UN vs. AMD B1-treated	37.98% 0.0012	1 ± 0.02236	0.6202 ± 0.03585
AMD UN vs. AMD B2-treated	38.43% 0.0012	1 ± 0.02236	0.6157 ± 0.02837
AMD UN vs. AMD B3-treated	35.83% 0.0022	1 ± 0.02236	0.6417 ± 0.02666
AMD UN vs. AMD B4-treated	22.68% 0.0022	1 ± 0.02236	0.7732 ± 0.03661
AMD UN vs. AMD B5-treated	31.93% 0.0022	1 ± 0.02236	0.6807 ± 0.03729
AMD UN vs. AMD B6-treated	30.79% 0.0022	1 ± 0.02236	0.6921 ± 0.02871

**Table 4:** Effects of resveratrol formulations on cell viability (**a**) and ROS levels (**b**) in AMD patient # 2.

**(a) AMD PATIENT #2_Resveratrol Effects on Cell Viability.**			
**Cell Viability**	**Percent Increase/*p*-Value**	**Untreated** **Mean ± SEM**	**Resveratrol Brands** **Mean ± SEM**
AMD UN vs. AMD B1-treated	25.5% 0.0159	1 ± 0.009554	1.255 ± 0.03819
AMD UN vs. AMD B2-treated	39% 0.0159	1 ± 0.009554	1.390 ± 0.1207
AMD UN vs. AMD B3-treated	9.2% 0.0357	1 ± 0.009554	1.092 ± 0.04765
AMD UN vs. AMD B4-treated	28% 0.0016	1 ± 0.009554	1.280 ± 0.03231
AMD UN vs. AMD B5-treated	25% 0.0025	1 ± 0.009554	1.250 ± 0.04042
AMD UN vs. AMD B6-treated	24.1% 0.0016	1 ± 0.009554	1.241 ± 0.03463
**(b) AMD PATIENT #2_Resveratrol Effects on ROS Levels.**			
**Ros Levels**	**Percent Decrease/*p*-Value**	**Untreated** **Mean ± SEM**	**Resveratrol Brands** **Mean ± SEM**
AMD UN vs. AMD B1-treated	56.1% 0.0002	1 ± 0.01954	0.4390 ± 0.01638
AMD UN vs. AMD B2-treated	59.08% 0.0002	1 ± 0.01954	0.4092 ± 0.02385
AMD UN vs. AMD B3-treated	45.86% 0.0002	1± 0.01954	0.5414 ± 0.03204
AMD UN vs. AMD B4-treated	18.26% 0.0040	1 ± 0.01954	0.8174 ± 0.06757
AMD UN vs. AMD B5-treated	24.81% 0.0002	1 ± 0.01954	0.7519 ± 0.03579
AMD UN vs. AMD B6-treated	21.63% 0.0040	1 ± 0.01954	0.7837 ± 0.01963

**Table 5:** Effects of resveratrol formulations on cell viability (**a**) and ROS levels (**b**) in AMD patient # 3.

**(a) AMD PATIENT #3_Resveratrol Effects on Cell Viability.**			
**Cell Viability**	**Percent Increase/*p*-Value**	**Untreated** **Mean ± SEM**	**Resveratrol Brands** **Mean ± SEM**
AMD UN vs. AMD B1-treated	31% 0.0003	1 ± 0.04315	1.310 ± 0.01742
AMD UN vs. AMD B2-treated	48% 0.0014	1 ± 0.04315	1.480 ± 0.02474
AMD UN vs. AMD B3-treated	57.9% 0.0014	1 ± 0.04315	1.579 ± 0.1443
AMD UN vs. AMD B4-treated	53.4% 0.0014	1 ± 0.04315	1.534 ± 0.02238
AMD UN vs. AMD B5-treated	42.7% 0.0014	1 ± 0.04315	1.427 ± 0.01969
AMD UN vs. AMD B6-treated	16.9% 0.0175	1 ± 0.04315	1.169 ± 0.01962
**(b) AMD PATIENT #3_Resveratrol Effects on ROS Levels.**			
**Ros Levels**	**Percent Decrease/*p*-Value**	**Untreated** **Mean ± SEM**	**Resveratrol Brands** **Mean ± SEM**
AMD UN vs. AMD B1-treated	66.68% 0.0014	1 ± 0.02114	0.3332 ± 0.01312
AMD UN vs. AMD B2-treated	49.79% 0.0014	1 ± 0.02114	0.5021 ± 0.008318
AMD UN vs. AMD B3-treated	54.96% 0.0014	1 ± 0.02114	0.4504 ± 0.01886
AMD UN vs. AMD B4-treated	19.68% 0.0021	1 ± 0.02114	0.8032 ± 0.03226
AMD UN vs. AMD B5-treated	4.17% 0.5074	1 ± 0.02114	0.9583 ± 0.04245
AMD UN vs. AMD B6-treated	17.2% 0.0294	1 ± 0.02114	0.8280 ± 0.04493

**Table 6:** Effects of resveratrol formulations on cell viability (**a**) and ROS levels (**b**) in AMD patient # 4.

**(a) AMD PATIENT #4_Resveratrol Effects on Cell Viability.**			
**Cell Viability**	**Percent Increase/*p*-Value**	**Untreated** **Mean ± SEM**	**Resveratrol Brands** **Mean ± SEM**
AMD UN vs. AMD B1-treated	25.1% 0.0294	1 ± 0.04158	1.251 ± 0.02404
AMD UN vs. AMD B2-treated	34.3% 0.0139	1 ± 0.04158	1.343 ± 0.04756
AMD UN vs. AMD B3-treated	18.1% 0.0498	1 ± 0.04158	1.181 ± 0.02298
AMD UN vs. AMD B4-treated	39.8% 0.0084	1 ± 0.04158	1.398 ± 0.03422
AMD UN vs. AMD B5-treated	14.8% 0.0195	1 ± 0.04158	1.148 ± 0.01154
AMD UN vs. AMD B6-treated	47.5% 0.0084	1 ± 0.04158	1.475 ± 0.02335
**(b) AMD PATIENT #4_Resveratrol Effects on ROS Levels.**			
**Ros Levels**	**Percent Decrease/*p*-Value**	**Untreated** **Mean ± SEM**	**Resveratrol Brands** **Mean ± SEM**
AMD UN vs. AMD B1-treated	55.75% 0.0043	1 ± 0.02706	0.4425 ± 0.01686
AMD UN vs. AMD B2-treated	56.32% 0.0043	1 ± 0.02706	0.4368 ± 0.02059
AMD UN vs. AMD B3-treated	50.5% 0.0079	1 ± 0.02706	0.4950 ± 0.04428
AMD UN vs. AMD B4-treated	14.99% 0.0714	1 ± 0.02706	0.8501 ± 0.06433
AMD UN vs. AMD B5-treated	36.55% 0.0079	1 ± 0.02706	0.6345 ± 0.04495
AMD UN vs. AMD B6-treated	26.44% 0.0079	1 ± 0.02706	0.7356 ± 0.04426

**Table 7:** Effects of resveratrol formulations on cell viability (**a**) and ROS levels (**b**) in AMD patient # 5.

**(a) AMD PATIENT #5_Resveratrol Effects on Cell Viability.**			
**Cell Viability**	**Percent Increase/*p*-Value**	**Untreated** **Mean ± SEM**	**Resveratrol Brands** **Mean ± SEM**
AMD UN vs. AMD B1-treated	42% 0.0021	1 ± 0.008937	1.420 ± 0.01545
AMD UN vs. AMD B2-treated	35.2% 0.0021	1 ± 0.008937	1.352 ± 0.02627
AMD UN vs. AMD B3-treated	23.9% 0.0021	1 ± 0.008937	1.239 ± 0.005641
AMD UN vs. AMD B4-treated	67% 0.0021	1 ± 0.008937	1.670 ± 0.01759
AMD UN vs. AMD B5-treated	23% 0.0021	1 ± 0.008937	1.230 ± 0.01031
AMD UN vs. AMD B6-treated	50.2% 0.0021	1 ± 0.008937	1.502 ± 0.05886
**(b) AMD PATIENT #5_Resveratrol Effects on ROS Levels.**			
**Ros Levels**	**Percent Decrease/*p*-Value**	**Untreated** **Mean ± SEM**	**Resveratrol Brands** **Mean ± SEM**
AMD UN vs. AMD B1-treated	40.36% 0.0012	1 ± 0.02983	0.5964 ± 0.02554
AMD UN vs. AMD B2-treated	43.83% 0.0012	1 ± 0.02983	0.5617 ± 0.01594
AMD UN vs. AMD B3-treated	32.1% 0.0022	1 ± 0.02983	0.6790 ± 0.04663
AMD UN vs. AMD B4-treated	1.6% 1.0000 *n*s	1 ± 0.02983	1.016 ± 0.01649
AMD UN vs. AMD B5-treated	20.35% 0.0238	1 ± 0.02983	0.7965 ± 0.008948
AMD UN vs. AMD B6-treated	16.39% 0.0238	1 ± 0.02983	0.8361 ± 0.003819

**Table 8:** Effects of resveratrol formulations on cell viability (**a**) and ROS levels (**b**) in AMD patient # 6.

**(a) AMD PATIENT #6_Resveratrol Effects on Cell Viability.**			
**Cell Viability**	**Percent Increase/*p*-Value**	**Untreated** **Mean ± SEM**	**Resveratrol brands** **Mean ± SEM**
AMD UN vs. AMD B1-treated	21.8% 0.0139	1 ± 0.02704	1.218 ± 0.03245
AMD UN vs. AMD B2-treated	11.3% 0.0498	1 ± 0.02704	1.113 ± 0.02333
AMD UN vs. AMD B3-treated	16.9% 0.0294	1 ± 0.02704	1.169 ± 0.04933
AMD UN vs. AMD B4-treated	47.9% 0.0084	1 ± 0.02704	1.479 ± 0.09846
AMD UN vs. AMD B5-treated	53.4% 0.0084	1 ± 0.02704	1.534 ± 0.08365
AMD UN vs. AMD B6-treated	47.4% 0.0106	1 ± 0.02704	1.474 ± 0.08017
**(b) AMD PATIENT #6_Resveratrol Effects on ROS Levels.**			
**Ros Levels**	**Percent Decrease/*p*-Value**	**Untreated** **Mean ± SEM**	**Resveratrol Brands** **Mean ± SEM**
AMD UN vs. AMD B1-treated	46.93% 0.0002	1 ± 0.05254	0.5307 ± 0.02323
AMD UN vs. AMD B2-treated	47.11% 0.0002	1 ± 0.05254	0.5289 ± 0.02670
AMD UN vs. AMD B3-treated	41.44% 0.0002	1 ± 0.05254	0.5856 ± 0.02532
AMD UN vs. AMD B4-treated	21.94% 0.0062	1 ± 0.05254	0.7806 ± 0.02889
AMD UN vs. AMD B5-treated	24.91% 0.0007	1 ± 0.05254	0.7509 ± 0.02245
AMD UN vs. AMD B6-treated	19.4% 0.0109	1 ± 0.05254	0.8060 ± 0.02580

**Table 9:** Effects of resveratrol formulations on cell viability (**a**) and ROS levels (**b**) in AMD patient # 7.

**(a) AMD PATIENT #7_Resveratrol Effects on Cell Viability.**			
**Cell Viability**	**Percent Increase/*p*-Value**	**Untreated** **Mean ± SEM**	**Resveratrol Brands** **Mean ± SEM**
AMD UN vs. AMD B1-treated	59.6% 0.0002	1 ± 0.01091	1.596 ± 0.02707
AMD UN vs. AMD B2-treated	47.5% 0.0002	1 ± 0.01091	1.475 ± 0.007203
AMD UN vs. AMD B3-treated	35.5% 0.0003	1 ± 0.01091	1.355 ± 0.01410
AMD UN vs. AMD B4-treated	75.6% 0.0003	1 ± 0.01091	1.756 ± 0.01699
AMD UN vs. AMD B5-treated	17.7% 0.0003	1 ± 0.01091	1.177 ± 0.007944
AMD UN vs. AMD B6-treated	48.8% 0.0003	1 ± 0.01091	1.488 ± 0.01817
**(b) AMD PATIENT #7_Resveratrol Effects on ROS Levels.**			
**Ros Levels**	**Percent Decrease/*p*-Value**	**Untreated** **Mean ± SEM**	**Resveratrol Brands** **Mean ± SEM**
AMD UN vs. AMD B1-treated	41.1% 0.0002	1 ± 0.05680	0.5890 ± 0.02433
AMD UN vs. AMD B2-treated	42.84% 0.0002	1 ± 0.05680	0.5716 ± 0.02857
AMD UN vs. AMD B3-treated	36.48% 0.0006	1 ± 0.05680	0.6352 ± 0.03928
AMD UN vs. AMD B4-treated	25.23% 0.0289	1 ± 0.05680	0.7477 ± 0.05962
AMD UN vs. AMD B5-treated	22.02% 0.0379	1 ± 0.05680	0.7798 ± 0.05155
AMD UN vs. AMD B6-treated	31.11% 0.0019	1 ± 0.05680	0.6889 ± 0.04688

**Table 10:** Effects of resveratrol formulations on cell viability (**a**) and ROS levels (**b**) in AMD patient # 8.

**(a) AMD PATIENT #8_Resveratrol Effects on Cell Viability.**			
**Cell Viability**	**Percent Increase/*p*-Value**	**Untreated** **Mean ± SEM**	**Resveratrol Brands** **Mean ± SEM**
AMD UN vs. AMD B1-treated	33% 0.0050	1 ± 0.05466	1.330 ± 0.07913
AMD UN vs. AMD B2-treated	28.6% 0.0286	1 ± 0.05466	1.286 ± 0.02095
AMD UN vs. AMD B3-treated	27.7% 0.0095	1 ± 0.05466	1.277 ± 0.006427
AMD UN vs. AMD B4-treated	38.2% 0.0040	1 ± 0.05466	1.382 ± 0.03517
AMD UN vs. AMD B5-treated	26.5% 0.0095	1 ± 0.05466	1.265 ± 0.02911
AMD UN vs. AMD B6-treated	0.8% 0.8000 ns	1 ± 0.05466	1.008 ± 0.002303
**(b) AMD PATIENT #8_Resveratrol Effects on ROS Levels.**			
**Ros Levels**	**Percent Decrease/*p*-Value**	**Untreated** **Mean ± SEM**	**Resveratrol Brands** **Mean ± SEM**
AMD UN vs. AMD B1-treated	69.13% 0.0003	1 ± 0.03698	0.3087 ± 0.007325
AMD UN vs. AMD B2-treated	63.83% 0.0003	1 ± 0.03698	0.3617 ± 0.01029
AMD UN vs. AMD B3-treated	44.76% 0.0003	1 ± 0.03698	0.5524 ± 0.02613
AMD UN vs. AMD B4-treated	17.06% 0.0047	1 ± 0.03698	0.8294 ± 0.02891
AMD UN vs. AMD B5-treated	13.5% 0.0303	1 ± 0.03698	0.8650 ± 0.03150
AMD UN vs. AMD B6-treated	28.33% 0.0006	1 ± 0.03698	0.7167 ± 0.01718

**Table 11:** Effects of resveratrol formulations on cell viability (**a**) and ROS levels (**b**) in AMD patient # 9.

**(a) AMD PATIENT #9_Resveratrol Effects on Cell Viability.**			
**Cell Viability**	**Percent Increase/*p*-Value**	**Untreated** **Mean ± SEM**	**Resveratrol Brands** **Mean ± SEM**
AMD UN vs. AMD B1-treated	61.1% 0.0025	1 ± 0.01018	1.611 ± 0.02128
AMD UN vs. AMD B2-treated	63% 0.0025	1 ± 0.01018	1.630 ± 0.06816
AMD UN vs. AMD B3-treated	37.8% 0.0025	1 ± 0.01018	1.378 ± 0.01167
AMD UN vs. AMD B4-treated	70.2% 0.0025	1 ± 0.01018	1.702 ± 0.07932
AMD UN vs. AMD B5-treated	21.9% 0.0025	1 ± 0.01018	1.219 ± 0.01320
AMD UN vs. AMD B6-treated	55.9% 0.0025	1 ± 0.01018	1.559 ± 0.04112
**(b) AMD PATIENT #9_Resveratrol Effects on ROS Levels.**			
**Ros Levels**	**Percent Decrease/*p*-Value**	**Untreated** **Mean ± SEM**	**Resveratrol Brands** **Mean ± SEM**
AMD UN vs. AMD B1-treated	18.01% 0.0242	1 ± 0.02608	0.8199 ± 0.04955
AMD UN vs. AMD B2-treated	24.31% 0.0061	1 ± 0.02608	0.7569 ± 0.03313
AMD UN vs. AMD B3-treated	17.15% 0.0095	1 ± 0.02608	0.8285 ± 0.02548
AMD UN vs. AMD B4-treated	13.57% 0.4000 *n*s	1 ± 0.02608	0.8643 ± 0.09676
AMD UN vs. AMD B5-treated	10.87% 0.4000 *n*s	1 ± 0.02608	0.8913 ± 0.06032
AMD UN vs. AMD B6-treated	18.38% 0.0159	1 ± 0.02608	0.8162 ± 0.03120

**Table 12:** Effects of resveratrol formulations on cell viability (**a**) and ROS levels (**b**) in AMD patient # 10.

**(a) AMD PATIENT #10_Resveratrol Effects on Cell Viability.**			
**Cell Viability**	**Percent Increase/*p*-Value**	**Untreated** **Mean ± SEM**	**Resveratrol Brands** **Mean ± SEM**
AMD UN vs. AMD B1-treated	50.9% 0.0002	1 ± 0.03038	1.509 ± 0.01864
AMD UN vs. AMD B2-treated	44.4% 0.0002	1 ± 0.03038	1.444 ± 0.03925
AMD UN vs. AMD B3-treated	31.4% 0.0002	1 ± 0.03038	1.314 ± 0.008565
AMD UN vs. AMD B4-treated	76.8% 0.0002	1 ± 0.03038	1.768 ± 0.03247
AMD UN vs. AMD B5-treated	32.7% 0.0002	1 ± 0.03038	1.327 ± 0.01818
AMD UN vs. AMD B6-treated	53% 0.0002	1 ± 0.03038	1.530 ± 0.06054
**(b) AMD PATIENT #10_Resveratrol Effects on ROS Levels.**			
**Ros Levels**	**Percent Decrease/*p*-Value**	**Untreated** **Mean ± SEM**	**Resveratrol Brands** **Mean ± SEM**
AMD UN vs. AMD B1-treated	39.69% 0.0006	1 ± 0.03429	0.6031 ± 0.02462
AMD UN vs. AMD B2-treated	37.53% 0.0006	1 ± 0.03429	0.6247 ± 0.02274
AMD UN vs. AMD B3-treated	30.69% 0.0006	1 ± 0.03429	0.6931 ± 0.02408
AMD UN vs. AMD B4-treated	6.9% 0.2667 *n*s	1 ± 0.03429	1.069 ± 0.02530
AMD UN vs. AMD B5-treated	24.92% 0.0006	1 ± 0.03429	0.7508 ± 0.02486
AMD UN vs. AMD B6-treated	13.47% 0.0262	1 ± 0.03429	0.8653 ± 0.03682

**Table 13:** Effects of resveratrol formulations on cell viability (**a**) and ROS levels (**b**) in AMD patient # 11.

**(a) AMD PATIENT #11_Resveratrol Effects on Cell Viability.**			
**Cell Viability**	**Percent Increase/*p*-Value**	**Untreated** **Mean ± SEM**	**Resveratrol Brands** **Mean ± SEM**
AMD UN vs. AMD B1-treated	203.4% 0.0034	1 ± 0.02849	3.034 ± 0.01950
AMD UN vs. AMD B2-treated	209% 0.0034	1 ± 0.02849	3.090 ± 0.1106
AMD UN vs. AMD B3-treated	165.9% 0.0034	1 ± 0.02849	2.659 ± 0.1311
AMD UN vs. AMD B4-treated	181.9% 0.0034	1 ± 0.02849	2.819 ± 0.01619
AMD UN vs. AMD B5-treated	61.8% 0.0034	1 ± 0.02849	1.618 ± 0.007562
AMD UN vs. AMD B6-treated	112.2% 0.0034	1 ± 0.02849	2.122 ± 0.03280
**(b) AMD PATIENT #11_Resveratrol Effects on ROS Levels.**			
**Ros Levels**	**Percent Decrease/*p*-Value**	**Untreated** **Mean ± SEM**	**Resveratrol Brands** **Mean ± SEM**
AMD UN vs. AMD B1-treated	32.84% 0.0002	1 ± 0.03538	0.6716 ± 0.04014
AMD UN vs. AMD B2-treated	37.66% 0.0002	1 ± 0.03538	0.6234 ± 0.04212
AMD UN vs. AMD B3-treated	34.71% 0.0002	1 ± 0.03538	0.6529 ± 0.03225
AMD UN vs. AMD B4-treated	33.18% 0.0002	1 ± 0.03538	0.6682 ± 0.02027
AMD UN vs. AMD B5-treated	30.16% 0.0002	1 ± 0.03538	0.6984 ± 0.02910
AMD UN vs. AMD B6-treated	33.61% 0.0002	1 ± 0.03538	0.6639 ± 0.03013

**Table 14:** Effects of resveratrol formulations on cell viability (**a**) and ROS levels (**b**) in AMD patient # 12.

**(a) AMD PATIENT #12_Resveratrol Effects on Cell Viability.**			
**Cell Viability**	**Percent Increase/*p*-Value**	**Untreated** **Mean ± SEM**	**Resveratrol Brands** **Mean ± SEM**
AMD UN vs. AMD B1-treated	57.3% 0.0005	1 ± 0.01253	1.573 ± 0.01362
AMD UN vs. AMD B2-treated	19.1% 0.0043	1 ± 0.01253	1.191 ± 0.05657
AMD UN vs. AMD B3-treated	17.3% 0.0095	1 ± 0.01253	1.173 ± 0.004108
AMD UN vs. AMD B4-treated	35.4% 0.0007	1 ± 0.01253	1.354 ± 0.03830
AMD UN vs. AMD B5-treated	6.6% 0.0095	1 ± 0.01253	1.066 ± 0.009232
AMD UN vs. AMD B6-treated	16.6% 0.0027	1 ± 0.01253	1.166 ± 0.04125
**(b) AMD PATIENT #12_Resveratrol Effects on ROS Levels.**			
**Ros Levels**	**Percent Decrease/*p*-Value**	**Untreated** **Mean ± SEM**	**Resveratrol Brands** **Mean ± SEM**
AMD UN vs. AMD B1-treated	63.04% 0.0001	1 ± 0.02391	0.3696 ± 0.01083
AMD UN vs. AMD B2-treated	56.68% 0.0009	1 ± 0.02391	0.4332 ± 0.02135
AMD UN vs. AMD B3-treated	26.04% 0.0013	1 ± 0.02391	0.7396 ± 0.04140
AMD UN vs. AMD B4-treated	11.05% 0.0100	1 ± 0.02391	0.8895 ± 0.02766
AMD UN vs. AMD B5-treated	19.12% 0.0019	1 ± 0.02391	0.8088 ± 0.05079
AMD UN vs. AMD B6-treated	11.33% 0.0317	1 ± 0.02391	0.8867 ± 0.004355

**Table 15:** Effects of resveratrol formulations on cell viability (**a**) and ROS levels (**b**) in AMD patient # 13.

**(a) AMD PATIENT #13_Resveratrol Effects on Cell Viability.**			
**Cell Viability**	**Percent Increase/*p*-Value**	**Untreated** **Mean ± SEM**	**Resveratrol Brands** **Mean ± SEM**
AMD UN vs. AMD B1-treated	84.3% 0.0002	1 ± 0.05060	1.843 ± 0.1106
AMD UN vs. AMD B2-treated	103.8% 0.0002	1 ± 0.05060	2.038 ± 0.1386
AMD UN vs. AMD B3-treated	57.6% 0.0002	1 ± 0.05060	1.576 ± 0.03683
AMD UN vs. AMD B4-treated	82.1% 0.0002	1 ± 0.05060	1.821 ± 0.09256
AMD UN vs. AMD B5-treated	36.6% 0.0012	1 ± 0.05060	1.366 ± 0.03996
AMD UN vs. AMD B6-treated	21% 0.0093	1 ± 0.05060	1.210 ± 0.02351
**(b) AMD PATIENT #13_Resveratrol Effects on ROS Levels.**			
**Ros Levels**	**Percent Decrease/*p*-Value**	**Untreated** **Mean ± SEM**	**Resveratrol Brands** **Mean ± SEM**
AMD UN vs. AMD B1-treated	66.14% 0.0007	1 ± 0.03609	0.3386 ± 0.01675
AMD UN vs. AMD B2-treated	51.29% 0.0007	1 ± 0.03609	0.4871 ± 0.01380
AMD UN vs. AMD B3-treated	49.99% 0.0007	1 ± 0.03609	0.5001 ± 0.02519
AMD UN vs. AMD B4-treated	22.14% 0.0007	1 ± 0.03609	0.7786 ± 0.04144
AMD UN vs. AMD B5-treated	25.98% 0.0043	1 ± 0.03609	0.7402 ± 0.05699
AMD UN vs. AMD B6-treated	31.56% 0.0007	1 ± 0.03609	0.6844 ± 0.04228

**Table 16:** Summary of the effects of resveratrol formulations on cell viability (**a**) and ROS levels (**b**) in all AMD patients # 1–13.

**(a) Summary of Resveratrol effects on Cell Viability.**													
**Treatment**	***P*1**	***P*2**	***P*3**	***P*4**	***P*5**	***P*6**	***P*7**	***P*8**	***P*9**	***P*10**	***P*11**	***P*12**	***P*13**
B1 Resveratrol Formulation	** 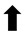	* 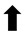	*** 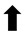	* 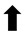	** 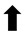	* 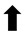	*** 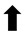	** 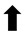	** 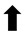	*** 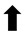	** 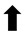	*** 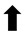	*** 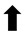
B2 Resveratrol Formulation	** 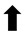	* 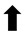	** 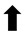	* 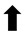	** 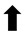	* 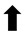	*** 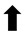	* 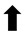	** 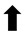	*** 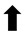	** 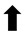	** 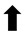	*** 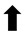
B3 Resveratrol Formulation	** 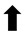	* 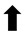	** 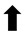	* 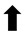	** 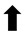	* 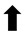	*** 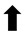	** 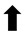	** 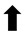	*** 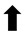	** 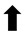	** 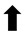	*** 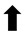
B4 Resveratrol Formulation	** 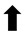	** 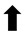	** 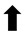	** 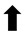	** 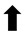	** 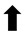	*** 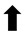	** 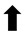	** 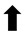	*** 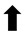	** 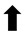	*** 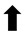	*** 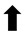
B5 Resveratrol Formulation	** 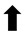	** 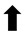	** 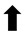	* 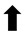	** 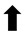	** 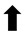	*** 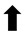	** 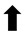	** 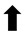	*** 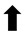	** 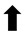	** 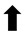	** 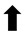
B6 Resveratrol Formulation	** 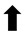	** 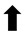	* 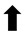	** 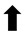	** 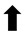	* 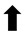	*** 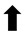	***n*s**	** 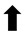	*** 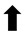	** 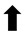	** 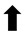	** 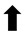
**(b) Summary of Resveratrol Effects on ROS Levels.**													
**Treatment**	***P*1**	***P*2**	***P*3**	***P*4**	***P*5**	***P*6**	***P*7**	***P*8**	***P*9**	***P*10**	***P*11**	***P*12**	***P*13**
B1 Resveratrol Formulation	** 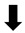	*** 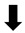	** 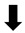	** 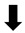	** 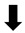	*** 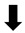	*** 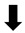	*** 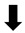	* 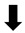	*** 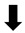	*** 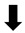	*** 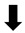	*** 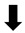
B2 Resveratrol Formulation	** 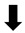	*** 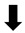	** 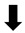	** 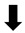	** 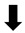	*** 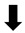	*** 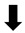	*** 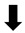	** 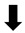	*** 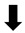	*** 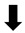	*** 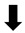	*** 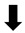
B3 Resveratrol Formulation	** 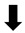	*** 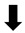	** 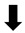	** 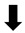	** 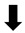	*** 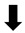	*** 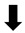	*** 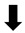	** 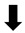	*** 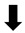	*** 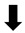	** 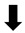	*** 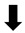
B4 Resveratrol Formulation	** 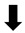	** 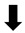	** 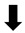	***n*s**	***n*s**	** 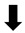	* 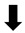	** 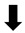	***n*s**	***n*s**	*** 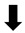	* 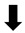	*** 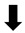
B5 Resveratrol Formulation	** 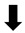	*** 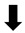	***n*s**	** 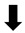	* 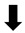	*** 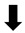	* 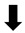	* 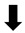	***n*s**	*** 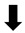	*** 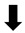	** 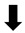	** 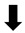
B6 Resveratrol Formulation	** 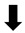	** 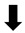	* 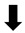	** 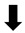	* 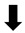	* 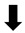	** 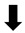	*** 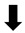	* 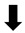	* 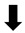	*** 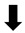	* 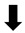	*** 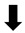

‘*n*s’ = non-significant; * = *p* < 0.05; ** = *p* < 0.01; *** = *p* < 0.001, 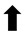 Increase; 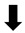 Decrease.
